# Assessing changes in incubation period, serial interval, and generation time of SARS-CoV-2 variants of concern: a systematic review and meta-analysis

**DOI:** 10.1186/s12916-023-03070-8

**Published:** 2023-09-29

**Authors:** Xiangyanyu Xu, Yanpeng Wu, Allisandra G. Kummer, Yuchen Zhao, Zexin Hu, Yan Wang, Hengcong Liu, Marco Ajelli, Hongjie Yu

**Affiliations:** 1 School of Public Health, Fudan University, Key Laboratory of Public Health Safety, Ministry of Education, Shanghai, China; 2 Shanghai Institute of Infectious Disease and Biosecurity, Fudan University, Shanghai, China; 3 Laboratory of Computational Epidemiology and Public Health, Department of Epidemiology and Biostatistics, Indiana University School of Public Health, Bloomington, IN USA

**Keywords:** COVID-19, Variants of concern, Incubation period, Serial interval, Realized generation time, Intrinsic generation time, Systematic review, Meta-analysis

## Abstract

**Background:**

After the first COVID-19 wave caused by the ancestral lineage, the pandemic has been fueled from the continuous emergence of new SARS-CoV-2 variants. Understanding key time-to-event periods for each emerging variant of concern is critical as it can provide insights into the future trajectory of the virus and help inform outbreak preparedness and response planning. Here, we aim to examine how the incubation period, serial interval, and generation time have changed from the ancestral SARS-CoV-2 lineage to different variants of concern.

**Methods:**

We conducted a systematic review and meta-analysis that synthesized the estimates of incubation period, serial interval, and generation time (both realized and intrinsic) for the ancestral lineage, Alpha, Beta, and Omicron variants of SARS-CoV-2.

**Results:**

Our study included 280 records obtained from 147 household studies, contact tracing studies, or studies where epidemiological links were known. With each emerging variant, we found a progressive shortening of each of the analyzed key time-to-event periods, although we did not find statistically significant differences between the Omicron subvariants. We found that Omicron BA.1 had the shortest pooled estimates for the incubation period (3.49 days, 95% CI: 3.13–4.86 days), Omicron BA.5 for the serial interval (2.37 days, 95% CI: 1.71–3.04 days), and Omicron BA.1 for the realized generation time (2.99 days, 95% CI: 2.48–3.49 days). Only one estimate for the intrinsic generation time was available for Omicron subvariants: 6.84 days (95% CrI: 5.72–8.60 days) for Omicron BA.1. The ancestral lineage had the highest pooled estimates for each investigated key time-to-event period. We also observed shorter pooled estimates for the serial interval compared to the incubation period across the virus lineages. When pooling the estimates across different virus lineages, we found considerable heterogeneities (*I*^2^ > 80%; *I*^2^ refers to the percentage of total variation across studies that is due to heterogeneity rather than chance), possibly resulting from heterogeneities between the different study populations (e.g., deployed interventions, social behavior, demographic characteristics).

**Conclusions:**

Our study supports the importance of conducting contact tracing and epidemiological investigations to monitor changes in SARS-CoV-2 transmission patterns. Our findings highlight a progressive shortening of the incubation period, serial interval, and generation time, which can lead to epidemics that spread faster, with larger peak incidence, and harder to control. We also consistently found a shorter serial interval than incubation period, suggesting that a key feature of SARS-CoV-2 is the potential for pre-symptomatic transmission. These observations are instrumental to plan for future COVID-19 waves.

**Supplementary Information:**

The online version contains supplementary material available at 10.1186/s12916-023-03070-8.

## Background

As of April 12, 2023, the COVID-19 pandemic has resulted in more than 762 million reported cases and 6.8 million reported deaths [1]. Since the first detection of SARS-CoV-2 (the virus causing COVID-19) in Wuhan, China, in December 2020, the virus has started to evolve, and several variants of SARS-CoV-2 have been identified. Five of them were classified as variants of concern (VOCs): Alpha (B.1.1.7), Beta (B.1.351), Gamma (P.1), Delta (B.1.617.2), and Omicron (B.1.1.529) [2]. Alpha and Delta were particularly successful at spreading around the globe, causing major waves of infections and associated hospitalizations since late 2020 and early 2021 [3, 4]. In late 2021, Omicron was first detected in South Africa and spread more rapidly than all other VOCs, causing massive outbreaks worldwide albeit with a lower associated burden due to high vaccination levels [2, 5, 6]. As of August 2023, Omicron subvariants are dominant worldwide.

**Fig. 1:**
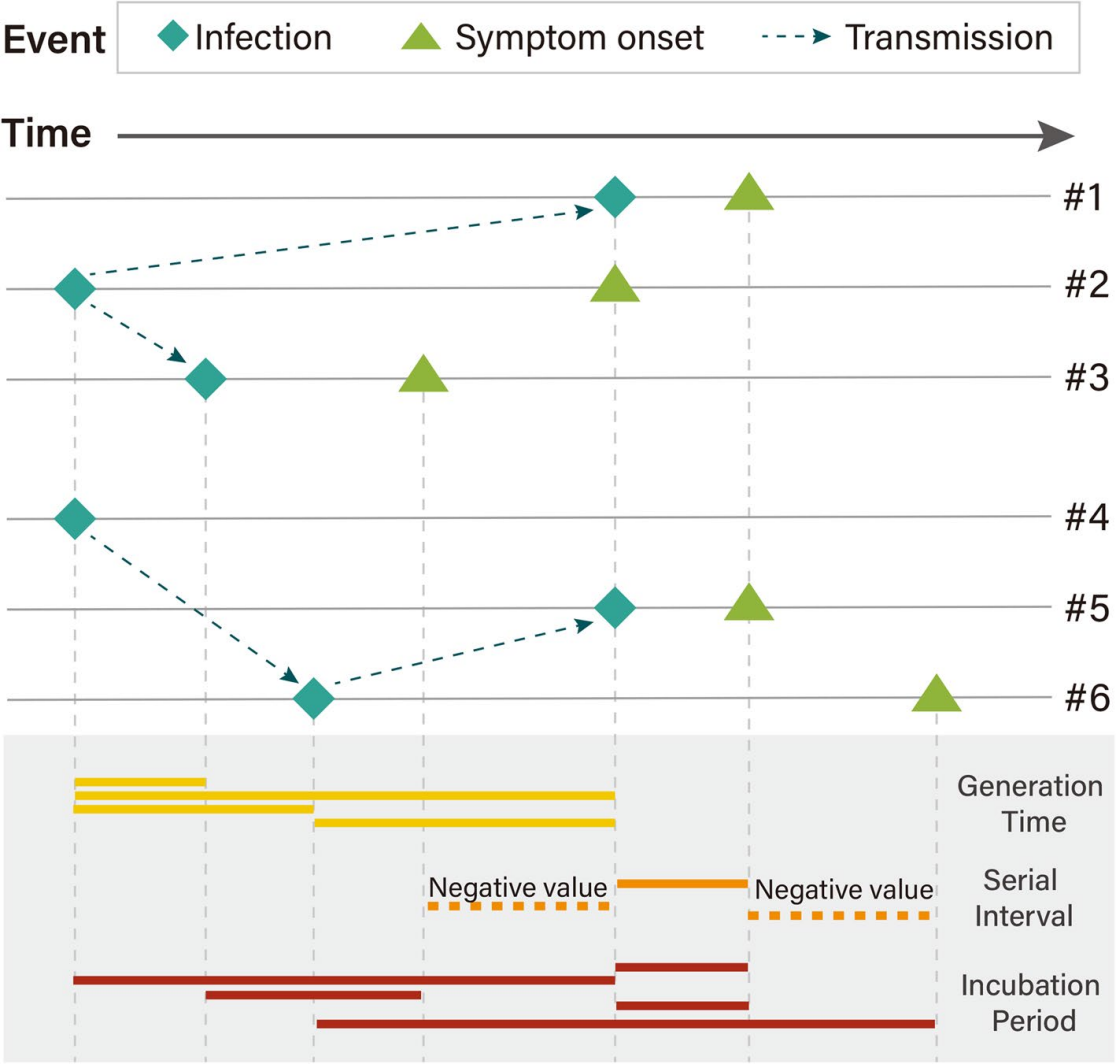
Illustrative example of individual observations of the incubation period, serial interval, and generation time. The top part of the figure shows two hypothetical transmission chains as well as the dates of infection and symptom onset (if any) of infected individuals. Each row corresponds to a study participant (#1–#6). The bottom part of the figure shows the estimated individual values of the incubation period, serial interval, and generation time

Knowledge of the transmission dynamics of SARS-CoV-2 VOCs is vital for understanding the epidemiology of COVID-19 and establishing effective control measures. In this context, three key indicators are the incubation period, serial interval, and generation time (Fig. 1). First, the incubation period is the interval between an individual’s time of infection and symptom onset; this indicator has crucial public health implications as, for instance, it can be used for (i) defining the length of the quarantine period and (ii) designing clinical trials (e.g., to monitor participants for disease signs after exposure). Second, the serial interval is the interval between symptom onset of the infector and symptom onset of the infectee(s); the serial interval is relevant, for example, for (i) defining contact tracing protocols (e.g., for determining where and from whom an individual may have contracted the pathogen anticipate who that person might have infected, and determining the timeframe for potential secondary infections) and (ii) evaluating the effectiveness of interventions (i.e., a lengthening serial interval might indicate reduced transmission, while a shortening or consistent serial interval may suggest ongoing transmission despite interventions). Third, the generation time is the interval between the infector’s time of infection and the infectee(s)’s time of infection; the generation time is crucial for (i) determining the speed of transmission of an epidemic outbreak (short generation time indicates rapid spread), and (ii) informing infectious disease models (as they rely on estimates of the generation time to simulate successive generations of infections). It is important to note that the generation time encompasses a non-linear combination of the latent period (time interval from infection to start of infectiousness) and infectious period (time interval from start of infectiousness to end of infectiousness) [7], as these two time periods may not be independent, and infectiousness can be variable over time [8, 9]. Moreover, it is important to stress that the distributions of the generation time and serial interval can be substantially different, especially in the presence of pre-symptomatic transmission, as it is the case for COVID-19. For instance, the distribution of the serial interval is generally symmetric and includes negative values; on the contrary, the distribution of the generation time is generally skewed, and the support of the distribution is strictly positive. Thus, it is crucial to have separate estimates of the distributions of the serial interval and generation time.

While many epidemiological investigations have estimated the incubation period, serial interval, and generation time for the VOCs, these indicators can be affected by a variety of factors related to the specific epidemiological situation of each study as well as deployed interventions [10–14]. Moreover, individual characteristics such as age, medical history, and immunity status can affect an individual's immune response to the virus, thereby affecting the length of the incubation period, viral shedding, and onset of disease [15, 16]. For these reasons, it is essential to combine these estimates to provide a more comprehensive picture of these indicators for the different VOCs. Previously, Du et al. reported that the Omicron variant had the shortest incubation period, followed by the Delta variant and ancestral lineage [17]. Additionally, they observed a shorter serial interval for both the Delta and Omicron variants compared to the ancestral lineage. Wu et al. observed a gradual decrease in the incubation period of COVID-19 from the Alpha variant to the Omicron variant with the evolution of mutant strains [16]. Madewell et al. observed that serial interval estimates for Delta and Omicron were shorter than ancestral SARS-CoV-2 variants, and more recent Omicron subvariants had even shorter serial intervals [18]. However, estimates need to be updated as more studies have been published since these meta-analyses have been conducted, especially regarding Omicron subvariants. Moreover, the previous meta-analyses did not include the generation time, nor did they analyze the co-evolution of the incubation period, serial interval, and generation time over different phases of the COVID-19 pandemic. As SARS-CoV-2 continues to mutate, systematically monitoring and comparing changes in these fundamental epidemiological indicators can provide insights into their possible future trajectory, thus providing invaluable insights for outbreak preparedness and response planning.

## Methods

### Search strategy

We conducted a systematic search following the Preferred Reporting Items for Systematic Reviews and Meta-Analyses (PRISMA) guideline (see PRISMA checklist) [19]. We searched for studies published in English on three peer-reviewed databases (PubMed, Embase, and Web of Science) and five preprint servers (medRxiv, bioRxiv, Europe PMC, SSRN, and arXiv) using predefined search terms (Additional file 1: Table S1). All searches were conducted on March 28, 2023.

### Inclusion and exclusion criteria

Studies were included if they satisfied the following criteria: (1) provided at least one summarized statistic (e.g., central tendency and dispersion) for the incubation period, serial interval, or generation time; (2) relied on data from a contact tracing study, a household study or a study where epidemiological links were known.

We excluded studies as follows: (1) were meta-analyses and reviews, study protocols, media news, commentaries, or the full text was unavailable (e.g., conference abstract); (2) SARS-CoV-2 variant/sub-variant or study period not reported; (3) carried out analysis with sample size less than five; (4) were non-human studies; (5) methods were not described.

### Outcome measures

The outcome variables were incubation period, serial interval, and generation time. We further divided the generation time into two categories: intrinsic and realized. The intrinsic generation time represents the generation time that would be observed in a fully connected infinitely large susceptible population in the absence of interventions and behavioral change. The realized generation time refers to the generation time that is observed in field condition and is thus affected by specific features of the study population (e.g., interventions, individual behaviors, analyzed social settings) [20–22].

### Data extraction

Study screening and data extraction were performed independently by authors YP.W. and X.X., and inconsistencies were reconciled together with a third author, Y.Z. For eligible studies, we extracted all summary statistics related to the outcomes of interest, including mean, median, interquartile range (IQR), range, standard deviation, quantiles, 95% confidence interval (CI), and 95% credible interval (CrI). In addition, we collected descriptive data on the authors, article’s title, journal, date of publication, study location (country or region), date of the study period, total number of study subjects, variant type, and methods used for estimating key time-to-event intervals.

### Quality assessment

Two authors (Z.H. and Y.Z.) independently carried out the quality assessment of the included literature. The quality assessment scale was adapted from the Newcastle–Ottawa quality assessment questions (Additional file 1: Table S2) [23]. Disagreements between the two reviewers were resolved together with a third author, X.X.

### Data analysis

We described the central tendency (mean or median) and dispersion measure (range, IQR, 95% CI, or 95% CrI) for incubation period, serial interval, and generation time for the different VOCs via a forest plot. For studies reporting both mean and median, we preferred the mean estimates since most studies reported mean. For dispersion measures, we preferred 95% CI/CrI, followed by IQR and range. If CI/CrI was not provided, but sample size, standard deviation, or parametric distribution were provided, the adjusted confidence interval was calculated using the following formula as in references [16, 17]:

$$95\mathrm{\% CI }=\mathrm{ mean}\pm 1.96\times \frac{sd}{\sqrt{n}}$$

Next, we conducted a meta-analysis for different SARS-CoV-2 lineages and subvariants of the Omicron variant. The random-effects models were employed for all the pooling analyses, as they are more robust for small sample sizes. For studies reporting only the median and its associated dispersion measure, the corresponding mean, and 95% CI were approximated using methods described in Luo et al. [24]. If a study did not include a measure of central tendency or dispersion, the study was included in the review, but not in the meta-analysis. These methods were implemented using R function *metagen* from package *meta* [25]. The pooled average estimates with 95% CI were shown in forest plots. Then, we used the Wilcoxon test to assess the significance of the differences between the incubation periods, serial intervals, and generation times of the ancestral lineage, Alpha, Delta, Omicron variants, and subvariants of Omicron.

Publication bias was assessed using a funnel plot and Egger’s test. A 2-sided *P* < 0.05 was considered statistically significant. All analyses were performed in R (version 4.1.0.). This review was not registered.

## Results

### Search results

**Fig. 2:**
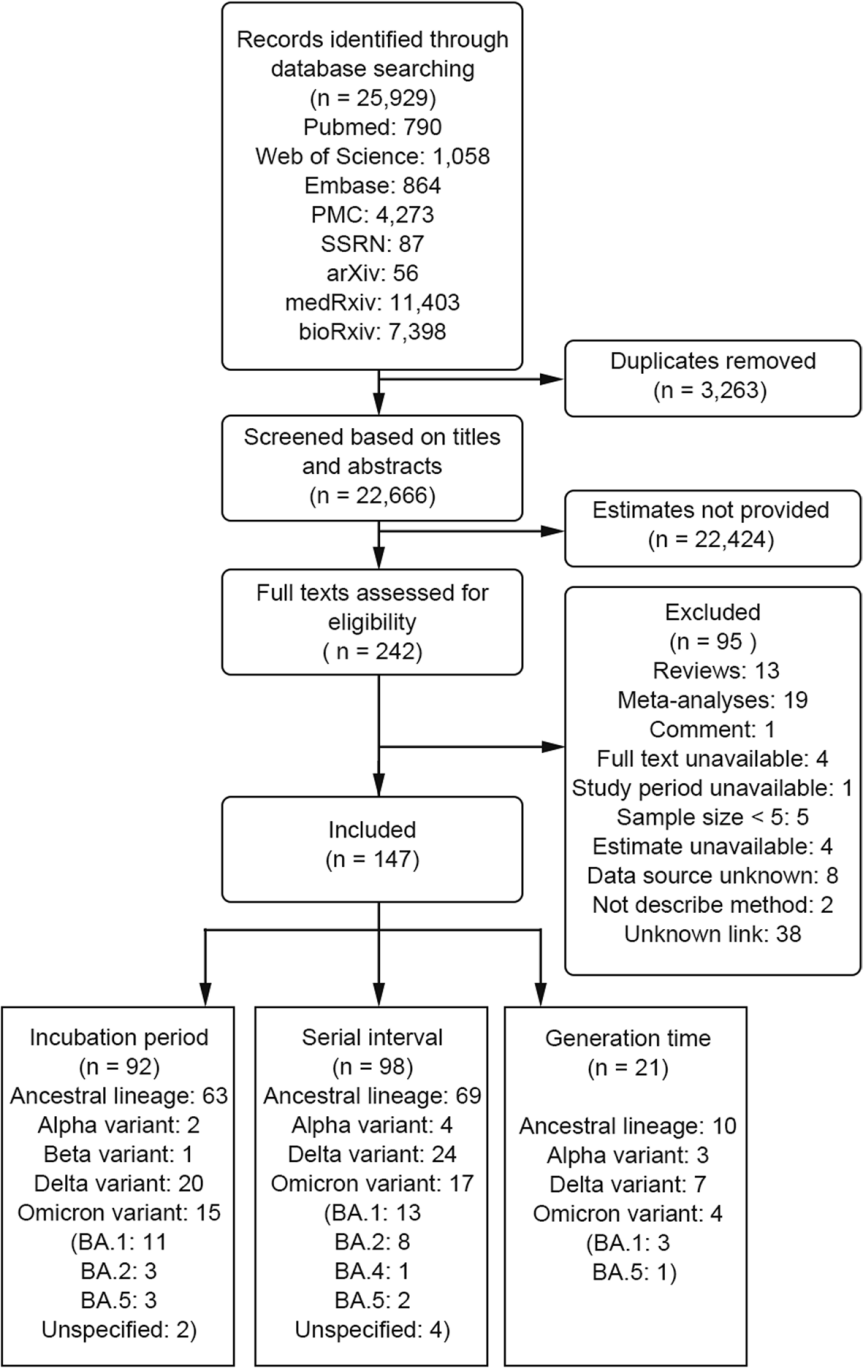
Study flow diagram

A total of 25,929 studies were identified based on our search strategy. After excluding 3,263 duplicated studies and an additional 22,424 articles via screening titles and abstracts, 242 articles were assessed for eligibility through a meticulous review of the full article. As per the inclusion/exclusion criteria, 147 studies were included in our analysis [9, 10, 12, 13, 21, 22, 26–166], 7 of which were from preprint platforms. The specific reasons for excluding other 95 studies can be found in Additional file 1: Table S3 [11, 16, 17, 23, 87, 167–256]. Among the included studies, 92 studies provided incubation period estimates, 98 studies provided serial interval estimates, and 21 studies provided generation time estimates (Fig. 2). Sixty-three studies reported more than one outcome; 18 studies provided estimates for more than one virus lineage.

### Study characteristics

We extracted 280 records for the focus key time-to-event periods from 147 included studies. Each record contained at least one summary statistic of central tendency and dispersion measure of the outcome variable(s). Three countries provided 11 records for the Alpha variant: 5 from Italy, 4 from the UK, 1 from Japan, and 1 from Germany. France was the only country to provide a record for the Beta variant. A total of 59 records were obtained for the Delta variant from 12 countries: 23 from China, 10 from the Netherlands, 5 from the UK, 4 from Italy, 4 from Japan, 4 from South Korea, 3 from Singapore, 2 from Spain, 1 from Germany, 1 from Belgium, 1 from Australia and 1 from Ireland. For Omicron BA.1, a total of 33 records were provided from 11 countries, including 10 from the Netherlands, 6 from China, 4 from Italy, 3 from South Korea, 2 from Japan, 2 from Singapore, 2 from Spain, 1 from the UK, 1 from Germany, 1 from Belgium and 1 from Norway. For Omicron BA.2, a total of 11 records were provided from 4 countries, including 8 from China, 1 from Singapore, 1 from the UK, and 1 from Germany. China was the only country to provide a record for Omicron BA.4. For Omicron BA.5, a total of 6 records were provided from 3 countries, including 4 from China, 1 from the UK, and 1 from Japan.

We identified 111 (39.6%) records for incubation period from 92 studies. Sixty-nine (62.2%) records focused on the ancestral lineage, 2 (1.8%) on Alpha variant, 1 (0.9%) on Beta variant, 20 (18.0%) on Delta variant, 11 (9.9%) on Omicron BA.1, 3 (2.7%) on Omicron BA.2 and 3 (2.7%) on Omicron BA.5.

For serial intervals, we obtained 137 (48.9%) records from 98 studies. Seventy-one (51.8%) records included focused on the ancestral lineage, 4 (2.9%) on Alpha variant, 29 (21.2%) on Delta variant, 17 (12.4%) on Omicron BA.1, 8 (5.8%) on Omicron BA.2, 1 (0.7%) on Omicron BA.4, and 2 (1.5%) on Omicron BA.5.

For the generation time, 32 (11.4%) records from 21 studies were included in our analysis, among them were 27 records for the realized generation time and 5 records for intrinsic generation time. For realized generation time, 11 (40.7%) studies focused on the ancestral lineage, 3 (11.1%) on Alpha variant, 8 (29.6%) on Delta variant, 4 (14.8%) on Omicron BA.1 and 1 (3.7%) on Omicron BA.5. For intrinsic generation time, 2 (40%) on Alpha variant, 2 (40%) on Delta variant, and 1 (20%) on Omicron BA.1.

Quality assessment (Additional file 1: Table S4 [9, 10, 12, 13, 21, 22, 26–32, 34, 37–40, 42–60, 62–65, 67–71, 73, 74, 76, 78–86, 89, 90, 92–94, 97–99, 101–107, 109–115, 117–121, 123–128, 130, 131, 133, 136–139, 141–153, 155–161, 164–166]) indicated that 1 study provided a precise exposure window for cases and identification of the potential infector(s). Sixty-eight studies included a well-characterized cohort of individuals that were comparable with the population and provided precise estimates for the symptom onset window for themselves and their potential infector(s).

### Incubation period

**Fig. 3:**
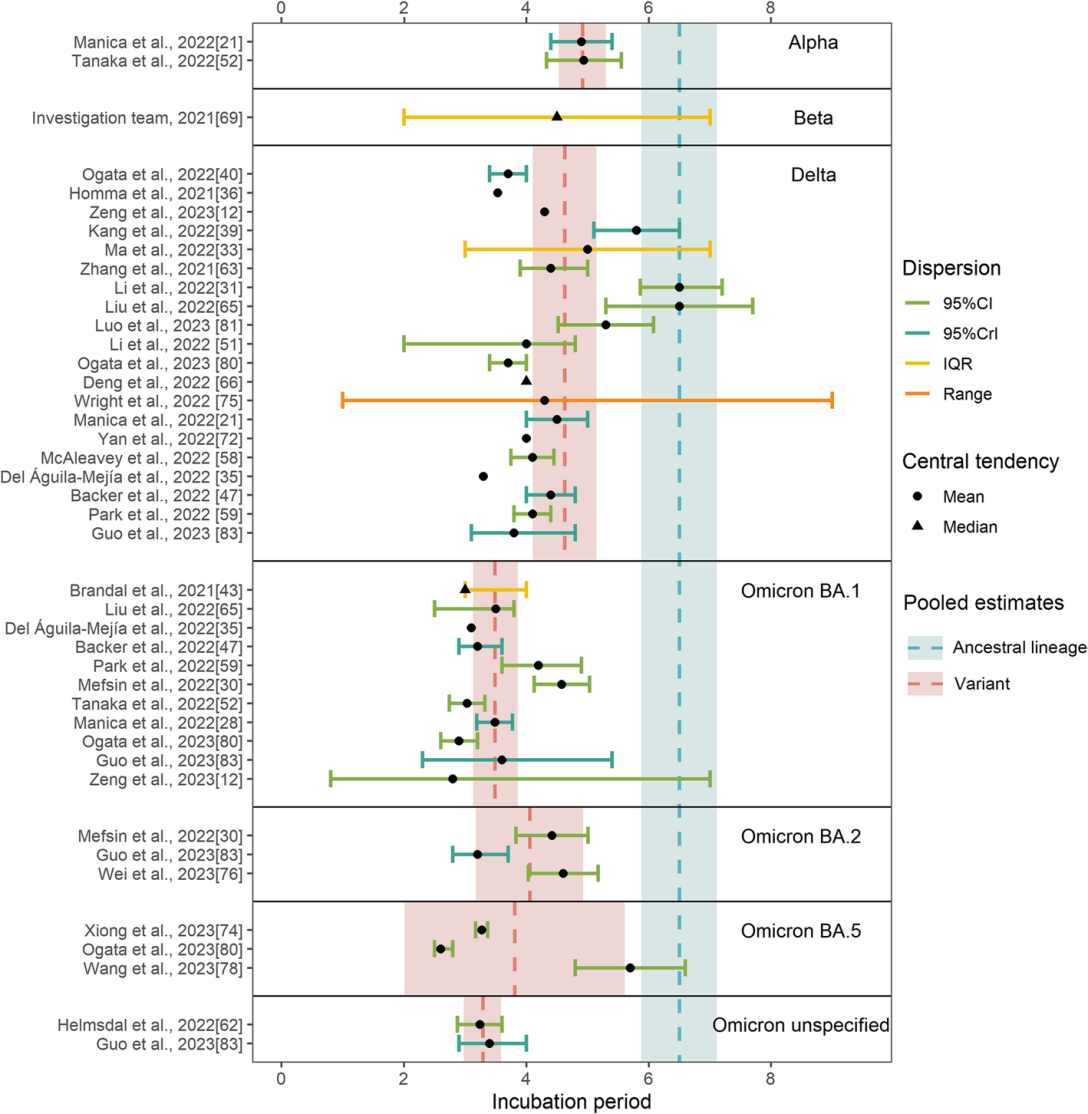
The reported estimates of the incubation period. The blue vertical dotted line and rectangle correspond to the pooled mean estimate and its 95% CI of the ancestral lineage, respectively. The red vertical dotted line and rectangle in different strata denote the pooled mean estimates and their 95% CI of corresponding variants, respectively. Black points and triangles represent mean estimates and median estimates, respectively. The horizontal segments indicate CI (green), CrI (light blue), IQR (yellow), and range (orange)

The ancestral lineage had the largest pooled mean incubation period (6.5 days, 95% CI: 5.88–7.12 days), followed by the Alpha variant (4.92 days, 95% CI: 4.53–5.30 days), Delta variant (4.63, 95% CI: 4.11–5.15 days), Omicron BA.2 (4.06 days, 95% CI: 3.18–4.93 days), Omicron BA.5 (3.81 days, 95% CI: 2.01–5.61 days), and Omicron BA.1 (3.49 days, 95% CI: 3.13–4.86 days) (Fig. 3, Additional file 1: Fig. S1 [9, 12, 21, 26, 28, 30–32, 34, 38–40, 42, 43, 47, 51, 52, 58–60, 62, 63, 65, 67, 70, 73, 74, 76, 78, 80, 81, 83, 85, 86, 92, 97, 98, 103–107, 109–115, 117–119, 130, 131, 133, 136–139, 142, 143, 155, 156, 165, 166]). Two studies did not specify the Omicron subvariant, and their pooled mean was 3.29 days (95% CI: 2.98–3.59 days); by pooling the estimates of the Omicron subvariants, the mean incubation period was 3.63 days (95% CI: 3.25–4.02 days). Only one study reported an estimate for the incubation period of the Beta variant (median: 4.5 days, IQR: 2–7 days). Delta, Omicron BA.1, Omicron BA.2 and Omicron BA.5 had significantly shorter incubation periods than the ancestral lineage (the *p*-values were 0.001, < 0.001, 0.023, and 0.050 for Delta, Omicron BA.1, Omicron BA.2, and Omicron BA.5, respectively; the sample sizes were 47, 14, 10, 3, and 3 for the ancestral lineage, Delta, Omicron BA.1, Omicron BA.2, and Omicron BA.5, respectively). Omicron BA.1 had significantly shorter incubation periods than Alpha and Delta (the *p*-values were 0.030 and 0.002 for Alpha and Delta, respectively). There was no significant difference between the other groups, which may be due to the small sample sizes. Our results suggested no potential publication bias in the included studies (*p*-value: 0.120) (Additional file 1: Fig. S2 [9, 12, 21, 26, 28, 30–32, 34, 38–40, 42, 43, 47, 51, 52, 58–60, 62, 63, 65, 67, 69, 70, 73, 74, 76, 78, 80, 81, 83, 85, 86, 92, 97, 98, 103–107, 109–115, 117–119, 130, 131, 133, 136–139, 142, 143, 155, 156, 165, 166]).

### Serial interval

**Fig. 4:**
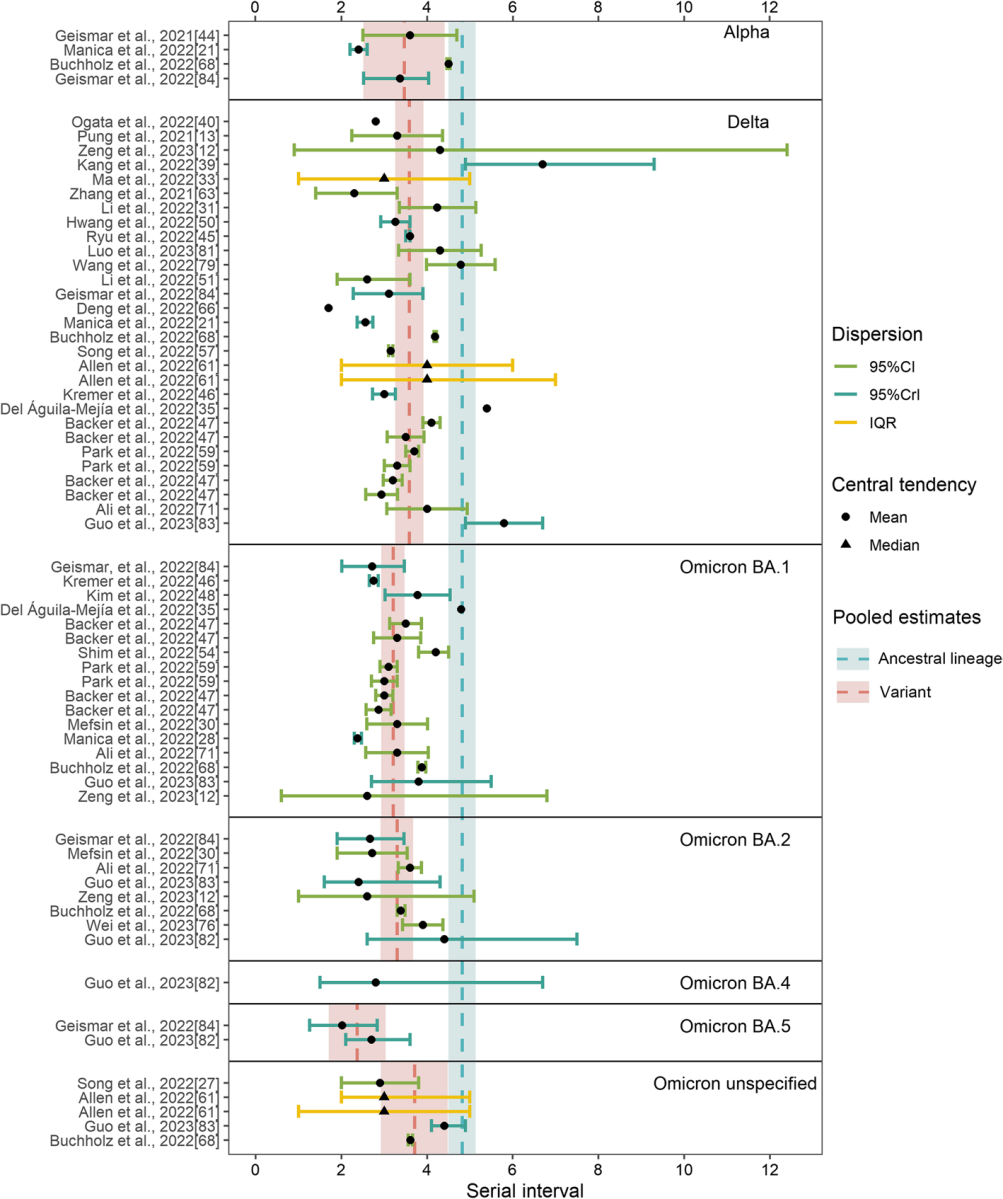
The reported estimates of the serial interval. The blue vertical dotted line and rectangle correspond to the pooled mean estimate and its 95% CI of the ancestral lineage, respectively. The red vertical dotted line and rectangle in different strata denote the pooled mean estimates and their 95% CI of corresponding variants, respectively. Black points and triangles represent mean estimates and median estimates, respectively. The horizontal segments indicate CI (green), CrI (light blue), and IQR (yellow)

The ancestral lineage had the largest pooled mean serial interval (4.82 days, 95% CI: 4.5–5.14 days), followed by the Delta variant (3.59 days, 95% CI: 3.26–3.92 days), Alpha variant (3.47 days, 95% CI: 2.52–4.41 days), Omicron BA.2 (3.3 days, 95% CI: 2.92–3.68 days), Omicron BA.1 (3.21 days, 95% CI: 2.94–3.48 days), and Omicron BA.5 (2.37 days, 95% CI: 1.71–3.04 days) (Fig. 4, Additional file 1: Fig. S3 [9, 10, 12, 13, 21, 27–31, 34, 37–39, 44–51, 53, 54, 56, 57, 59, 60, 63, 64, 67, 68, 71, 76, 79, 81–84, 89, 90, 93, 94, 98, 99, 106, 109, 110, 119–121, 123, 124, 126–128, 131, 133, 136–139, 141, 142, 144–153, 156–158, 160, 161, 164–166]). Five studies did not specify the Omicron subvariant, and their pooled mean was 3.71 days (95% CI: 2.93–4.49 days); by pooling the estimates of the Omicron subvariants, the mean incubation period was 3.19 days (95% CI: 2.95–3.43 days). A significantly shorter serial interval was found for each lineage compared to the ancestral lineage (*p*-values: 0.024, < 0.001, < 0.001, < 0.001, 0.026 for Alpha, Delta, Omicron BA.1, Omicron BA.2, and Omicron BA.5, respectively; the sample sizes were 60, 4, 23, 16, 8, and 2 for the ancestral lineage, Alpha, Delta, Omicron BA.1, Omicron BA.2, and Omicron BA.5, respectively). There was no significant difference between the other groups. Our results suggested no potential publication bias in the included studies (*p*-value: 0.700) (Additional file 1: Fig. S4 [9, 10, 12, 13, 21, 27–31, 34, 37–39, 44–51, 53, 54, 56, 57, 59, 60, 63, 64, 67, 68, 71, 76, 79, 81–84, 89, 90, 93, 94, 98, 99, 106, 109, 110, 119–121, 123, 124, 126–128, 131, 133, 136–139, 141, 142, 144–153, 156–158, 160, 161, 164–166]).

### Generation time

**Fig. 5:**
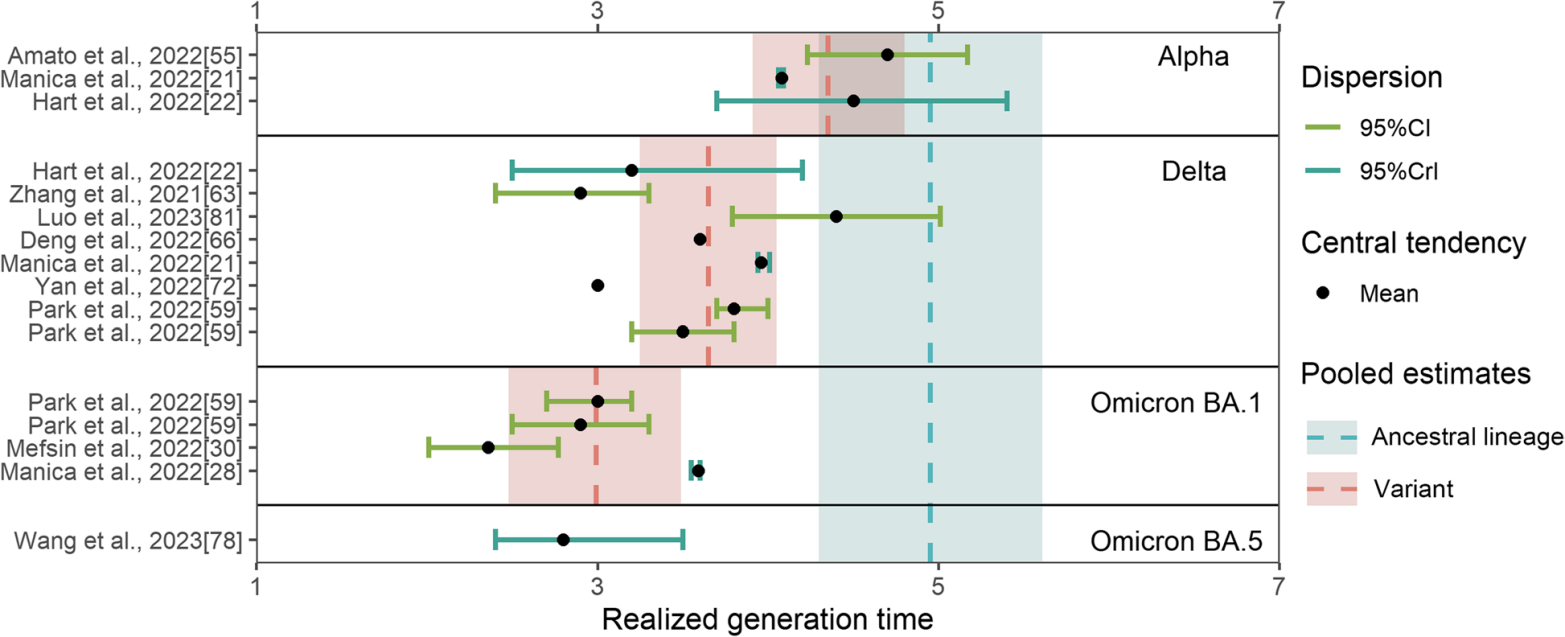
The reported estimates of the realized generation time. The blue vertical dotted line and rectangle correspond to the pooled mean estimate and its 95% CI of the ancestral lineage, respectively. The red vertical dotted line and rectangle in different strata denote the pooled mean estimates and their 95% CI of corresponding variants, respectively. Black points and triangles represent mean estimates and median estimates, respectively. The horizontal segments indicate CI (green) and CrI (light blue)

For the realized generation time, the ancestral lineage had the largest pooled mean generation time (4.95 days, 95% CI: 4.3–5.61 days), followed by the Alpha variant (4.35 days, 95% CI: 3.91–4.8 days), Delta variant (3.65 days, 95% CI: 3.25–4.05 days), and Omicron BA.1 (2.99 days, 95% CI: 2.48–3.49 days) (Fig. 5, Additional file 1: Fig. S5 [9, 21, 22, 28, 30, 55, 59, 63, 81, 101–104, 125, 142, 159]). Regarding the Omicron variant of all subvariants, the mean realized generation time was estimated to be 2.96 days (95% CI: 2.54–3.38 days). The realized generation times for the Delta and Omicron BA.1 were significantly shorter than the ancestral lineage (*p*-values: 0.021, 0.011 for Delta and Omicron BA.1, respectively; the sample sizes were 9, 6, 4 for the ancestral lineage, Delta, and Omicron BA.1, respectively). The central tendency of the realized generation time subsequently decreased by 16.1% from Alpha to Delta (*p*-value: 0.048), and there was no significant difference between the ancestral lineage and Alpha (*p*-value: 0.354; the sample size was 3 for Alpha), Alpha and Omicron BA.1 (*p*-value: 0.057), and Delta and Omicron BA.1 (*p*-value: 0.134). Our results suggested no potential publication bias in the included studies (*p*-value: 0.806) (Additional file 1: Fig. S6 [9, 21, 22, 28, 30, 55, 59, 63, 78, 81, 101–104, 125, 142, 159]).

**Fig. 6:**
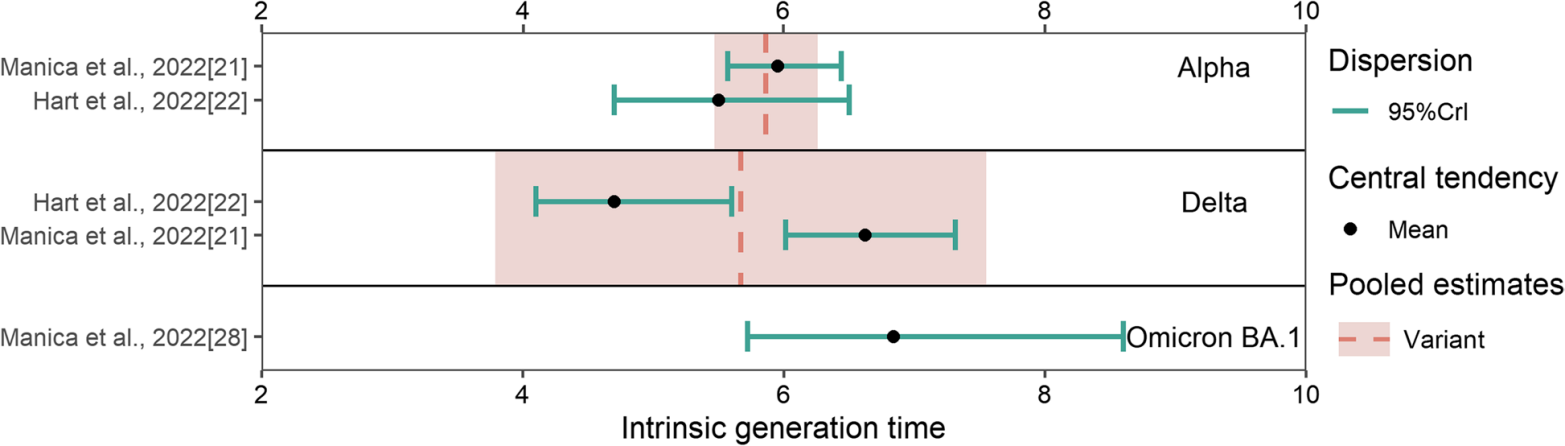
The reported estimates of the intrinsic generation time. The red vertical dotted line and rectangle in different strata denote the pooled mean estimates and their 95% CI of corresponding variants, respectively. Black points and triangles represent mean estimates and median estimates, respectively. The horizontal segments indicate CrI (light blue)

For the intrinsic generation time, we obtained a pooled mean estimate of 5.86 days (95% CI: 5.47–6.26 days) for the Alpha variant based on two studies and 5.67 days (95% CI: 3.79–7.55 days) for the Delta variant based on two studies (Fig. 6, Additional file 1: Fig. S7 [21, 22]). Only one study (for Italy) reported an estimate for the Omicron variant: 6.84 days (95% CrI: 5.72–8.60 days). There was no significant difference between Alpha and Delta (*p*-value: 1).

## Discussion

Our study revealed a progressive shortening of each of the analyzed key time-to-event periods, although we did not find statistically significant differences between the Omicron subvariants. We found that Omicron BA.1 had the shortest pooled estimates for the incubation period (3.49 days, 95% CI: 3.13–4.86 days), Omicron BA.5 for the serial interval (2.37 days, 95% CI: 1.71–3.04 days), and Omicron BA.1 for the realized generation time (2.99 days, 95% CI: 2.48–3.49 days). Only one estimate for the intrinsic generation time was available for Omicron subvariants: 6.84 days (95% CrI: 5.72–8.60 days) for Omicron BA.1. The ancestral lineage had the highest pooled estimates for each investigated key time-to-event period. Our results were comparable to those of Galmiche et al. who reported SARS-CoV-2 incubation period was notably reduced in omicron cases compared with all other variants of concern [257]. These findings suggest that the incubation period, serial interval, and realized generation times of COVID-19 became shorter over the course of the pandemic.

The majority of the studies included in our analysis provided estimates for the incubation period (92 studies) and serial interval (98 studies), while only 21 studies provided estimates for the generation time. This suggests that estimates for serial interval and incubation period are easier to obtain as they can more easily inferred from contact tracing and household studies than estimates for generation time, which requires more complex Bayesian analyses as the date of infection of the infector and their infectees are both generally unknown [21, 28]. Furthermore, China provided more records of estimates for the incubation period (60.6%), serial interval (34.6%), and generation time (37.5%) than any other country. This may be due to the smaller outbreak size of COVID-19 outbreaks in China before the rise of Omicron as compared to countries with widespread COVID-19 transmission, which made contact tracing and household studies easier to be conducted. This further supports the importance of contact tracing not only as a tool for monitoring and controlling infectious disease spread, but also for understanding transmission patterns.

Examining the intrinsic generation time is done using recently developed Bayesian methods, which may explain why only two studies have provided estimates for this indicator [22, 28]. Between these studies, there were only 5 records: 2 for Alpha, 2 the Delta, and 1 for Omicron BA.1. Given the small sample size, a pooled mean estimate was not warranted, and we could not compare possible differences between subsequent VOCs.

The incubation period of the ancestral lineage, and the Alpha, Beta, and Delta variants, is generally longer than that of other acute respiratory viral infections, such as human coronavirus (3.2 days), influenza A (1.43–1.64 days), parainfluenza (2.6 days), respiratory syncytial virus (4.4 days), and rhinovirus (1.4 days) [258]. Our findings produced similar mean incubation period estimates to those reported by Du et al. for Delta (4.8 days 95% CI: 3.9–5.6) and Omicron (3.6 days, 95% CI: 2.3–4.9) [17] and those reported by Wu et al. for Alpha (5.00 days, 95% CI: 4.94–5.06 days), Delta (4.41 days, 95% CI: 3.76–5.05 days), and Omicron (3.42 days, 95% CI: 2.88–3.96 days) [16].

Our study showed that the mean serial interval of COVID-19 ranged from 1.7 to 7.5 days. These estimates are longer than those of influenza A(H3N2) (2.2 days) and pandemic influenza A(H1N1)pdm09 (2.8 days), but shorter than those of the respiratory syncytial virus (RSV, 7.5 days), measles (11.7 days), varicella (14.0 days), smallpox (17.7 days), mumps (18.0 days), rubella (18.3 days), pertussis (22.8 days), and Middle East respiratory syndrome (MERS, 7.6 days) [259, 260]. Our results were comparable to those of Du et al. who reported an average serial interval of 3.4 days (95%CI: 3.0-3.7) for the Delta variant and 3.1 days (95%CI: 2.9-3.2) for the Omicron variant, respectively [17]. The mean generation time of COVID-19 ranged from 2.36 to 6.84 days, longer than those of influenza A (H1N1) (2 days) and pandemic influenza A(H1N1)pdm09 (2.92 days) [261], but shorter than those of the MERS (10.7 days) [262, 263].

Our estimates are consistent with previous estimates for the incubation period and serial interval. However, despite more stringent inclusion criteria, our study conducted a more comprehensive database search than Du et al. [17] and Wu et al. [16] and analyzed more VOCs than Madewell et al. [18]. Our findings provide updated estimates, as they include both more recent and a greater number of estimates than were included in previous studies (Additional file 1: Table S5 [16–18]). Furthermore, previous reviews have not provided estimates for generation time, which is further stratified into intrinsic and realized generation time. Finally, by focusing on three time-to-key-event periods, our study provides a systematic comparison of how these mutually associated quantities evolved throughout the COVID-19 pandemic.

Overall, the serial interval maintained a shorter pooled mean estimate compared to the incubation period across different virus lineages, indicating that a large proportion of SARS-CoV-2 transmission occurs prior to symptom onset [180, 216]. Pre-symptomatic transmission was a critical factor facilitating SARS-CoV-2 spread [264], highlighting the importance of obtaining timely estimates of these indicators and keep monitoring their possible changes over the course of an epidemic.

The current study provides scientific evidence that the incubation period of COVID-19 has shortened as the virus has evolved, which has important implications for the formulation of effective epidemic control strategies such as isolation and quarantine. Vaccination has been shown to lead to reduced viral loads and duration of shedding of SARS-CoV-2, which varies with waning immunity [265]. Virus replication and shedding abilities are variant specific, which impact the duration of the incubation period [265, 266]. At the individual level, vaccination or infection status can affect the immune response to the virus, which would impact the estimates of the incubation period across different viral lineages [267, 268]. Furthermore, vaccination coverage and vaccine products vary between countries, which may impact the estimates of the incubation period for different study sites for the same viral lineage [269]. More research is needed to quantify the extent to which immunity affects the incubation period over time with consideration for individual-level heterogeneities as well as the effects of country-level heterogeneities of immunity on the incubation period for each VOC.

Our findings also demonstrated that the serial intervals of COVID-19 shortened with each new VOC. Previous studies have attributed decreased serial intervals to preventive measures that target the duration of potential transmission, such as isolation, contact tracing, quarantine, and other non-pharmaceutical interventions (NPIs) [11–14], which is further supported by the fact that longer serial intervals are often censored due to case isolations [31]. The highly heterogeneous implementation of NPIs between countries, within country, and time frame has probably contributed to heterogeneous estimates of the serial interval that we observed between studies. This applies also for estimates of the realized generation time.

We acknowledge some limitations in this study. When pooling the estimates across different virus lineages, we found considerable heterogeneities (*I*^2^ > 80%; *I*^2^ refers to the percentage of total variation across studies that is due to heterogeneity rather than chance), possibly resulting from heterogeneities between the different study populations (e.g., deployed interventions, social behavior, demographic characteristics). Specifically, heterogeneities for the incubation period may be due to heterogeneities in the age structure and presence of pre-existing conditions in the host population [225, 270], whereas diversity in contact settings and the strength of NPIs may be responsible for heterogeneity of the estimates for the serial interval and realized generation time. Our analysis does not explore the observed heterogeneities because of the lack of harmonized data and information in the studies included in our meta-analysis. However, to incorporate these heterogeneities into the final estimates, we used random-effects models, as recommended by Deeks et al. [271]. This study may be limited by recall bias as many studies included in the analysis rely on retrospective data collection for exposure and symptom onset, which would influence the obtained estimates. The incubation period for the Beta variant and the intrinsic generation time for Omicron were each included in only one study, and thus it was not possible to produce pooled estimates. Moreover, our analysis may be subject to bias due to the inclusion of studies from the early phase of the pandemic, which may potentially lead to an underestimation of the incubation period, as suggested by Xin et al. [193].

## Conclusions

Our findings suggest that the incubation period, serial interval, and generation time of SARS-CoV-2 have evolved to shorter intervals with the emergence of each new VOC. Identifying the length of each of these indicators is critical for understanding the epidemiology of different SARS-CoV-2 variants and developing control measures for mitigating the spread of COVID-19. Moreover, understanding trends in these indicators can be instrumental for preparedness planning for future COVID-19 outbreaks.

### Supplementary Information

**Additional file 1: Table S1.** Search strategy and search results. **Table S2.** Quality assessment scale. **Table S3.** Excluded studies and reason for exclusion. **Table S4.** Quality assessment of the studies used in the meta-analysis. **Figure S1.** Forest plot for studies of reporting estimates of the incubation period for different SARS-CoV-2 lineages. **Figure S2.** Funnel plot for the incubation period with a 95% CI for studies included in the meta-analysis. **Figure S3.** Forest plot for studies of reporting estimates of the serial interval for different SARS-CoV-2 lineages. **Figure S4.** Funnel plot for the serial interval with a 95% CI for studies included in the meta-analysis. **Figure S5.** Forest plot for studies of reporting estimates of the realized generation time for different SARS-CoV-2 lineages. **Figure S6.** Funnel plot for the realized generation time with a 95% CI for studies included in the meta-analysis. **Figure S7.** Forest plot for studies of reporting estimates of the intrinsic generation time for different SARS-CoV-2 lineages. **Table S5.** Characteristics of previous meta-analyses of the incubation period and/or serial interval of SARS-CoV-2 VOCs.

## Data Availability

The dataset generated and analyzed in the current study are available online at https://github.com/xxyy0574/COVID-19-transmission-parameters.

## Abbreviations

CI: Confidence interval
CrI: Credible interval
IQR: Interquartile range
VOC: Variant of concern

## References

[1] WHO Coronavirus (COVID-19) Dashboard. https://covid19.who.int/. Accessed 20 April 2023.

[2] Tracking SARS-CoV-2 variants. https://www.who.int/activities/tracking-SARS-CoV-2-variants/. Accessed 20 April 2023.

[3] Salvatore M, Purkayastha S, Ganapathi L, Bhattacharyya R, Kundu R, Zimmermann L, et al. Lessons from SARS-CoV-2 in India: a data-driven framework for pandemic resilience. Sci Adv. 2022;8(24):eabp8621. 10.1126/sciadv.abp8621 PMC9205583 35714183

[4] Bolze A, Luo S, White S, Cirulli ET, Wyman D, Dei Rossi A, et al. SARS-CoV-2 variant Delta rapidly displaced variant Alpha in the United States and led to higher viral loads. Cell Rep Med. 2022;3(3):100564. 10.1016/j.xcrm.2022.100564 PMC8922438 35474739

[5] Aleem A, Akbar Samad AB, Vaqar S (2023). Emerging Variants of SARS-CoV-2 And Novel Therapeutics Against Coronavirus (COVID-19).

[6] Zhang L, Li Q, Liang Z, Li T, Liu S, Cui Q (2022). The significant immune escape of pseudotyped SARS-CoV-2 variant Omicron. Emerg Microbes Infect.

[7] Wallinga J, Lipsitch M (2007). How generation intervals shape the relationship between growth rates and reproductive numbers. Proc Biol Sci.

[8] He X, Lau EHY, Wu P, Deng X, Wang J, Hao X (2020). Temporal dynamics in viral shedding and transmissibility of COVID-19. Nat Med.

[9] Hu S, Wang W, Wang Y, Litvinova M, Luo K, Ren L (2021). Infectivity, susceptibility, and risk factors associated with SARS-CoV-2 transmission under intensive contact tracing in Hunan, China. Nat Commun.

[10] Ali ST, Wang L, Lau EHY, Xu X-K, Du Z, Wu Y (2020). Serial interval of SARS-CoV-2 was shortened over time by nonpharmaceutical interventions. Science.

[11] Ali ST, Yeung A, Shan S, Wang L, Gao H, Du Z (2022). Serial Intervals and Case Isolation Delays for Coronavirus Disease 2019: A Systematic Review and Meta-Analysis. Clin Infect Dis.

[12] Zeng K, Santhya S, Soong A, Malhotra N, Pushparajah D, Thoon KC, et al. Serial intervals and incubation periods of SARS-CoV-2 Omicron and Delta variants, Singapore. Emerg Infect Dis. 2023;29(4):814. 10.3201/eid2904.220854 PMC10045676 36878009

[13] Pung R, Mak TM, Kucharski AJ, Lee VJ (2021). Serial intervals in SARS-CoV-2 B.1.617.2 variant cases. Lancet..

[14] Ahlers M, Aralis H, Tang W, Sussman JB, Fonarow GC, Ziaeian B. Non-pharmaceutical interventions and covid-19 burden in the United States: retrospective, observational cohort study. BMJ Med. 2022;1(1):e000030. 10.1136/bmjmed-2021-000030 PMC9978758 36936598

[15] Hermesh T, Moltedo B, López CB, Moran TM (2010). Buying time-the immune system determinants of the incubation period to respiratory viruses. Viruses.

[16] Wu Y, Kang L, Guo Z, Liu J, Liu M, Liang W. Incubation period of COVID-19 caused by unique SARS-CoV-2 strains: a systematic review and meta-analysis. JAMA Netw Open. 2022;5(8):e2228008. 10.1001/jamanetworkopen.2022.28008 PMC9396366 35994285

[17] Du Z, Liu C, Wang L, Bai Y, Lau EHY, Wu P, et al. Shorter serial intervals and incubation periods in SARS-CoV-2 variants than the SARS-CoV-2 ancestral strain. J Travel Med. 2022;29(6):taac052. 10.1093/jtm/taac052 PMC9047226 35442440

[18] Madewell ZJ, Yang Y, Longini IM, Jr., Halloran ME, Vespignani A, Dean NE. Rapid review and meta-analysis of serial intervals for SARS-CoV-2 Delta and Omicron variants. BMC Infect Dis. 2023;23(1):429. 10.1186/s12879-023-08407-5 PMC10291789 37365505

[19] Page MJ, McKenzie JE, Bossuyt PM, Boutron I, Hoffmann TC, Mulrow CD, et al. The PRISMA 2020 statement: an updated guideline for reporting systematic reviews. BMJ. 2021;372:n71. 10.1136/bmj.n71 PMC8005924 33782057

[20] Champredon D, Dushoff J. Intrinsic and realized generation intervals in infectious-disease transmission. Proc Biol Sci. 2015;282(1821):20152026. 10.1098/rspb.2015.2026 PMC4707754 26674948

[21] Manica M, Litvinova M, De Bellis A, Guzzetta G, Mancuso P, Vicentini M, et al. Estimation of the incubation period and generation time of SARS-CoV-2 Alpha and Delta variants from contact tracing data. Epidemiol Infect. 2023;151:e5. 10.1017/S0950268822001947 PMC9837419 36524247

[22] Hart WS, Miller E, Andrews NJ, Waight P, Maini PK, Funk S, et al. Generation time of the alpha and delta SARS-CoV-2 variants: an epidemiological analysis. Lancet Infect Dis. 2022;22(5):603–10. 10.1016/S1473-3099(22)00001-9 PMC8843191 35176230

[23] McAloon C, Collins A, Hunt K, Barber A, Byrne AW, Butler F, et al. Incubation period of COVID-19: a rapid systematic review and meta-analysis of observational research. BMJ Open. 2020;10(8):e039652. 10.1136/bmjopen-2020-039652 PMC7430485 32801208

[24] Luo D, Wan X, Liu J, Tong T (2018). Optimally estimating the sample mean from the sample size, median, mid-range, and/or mid-quartile range. Stat Methods Med Res.

[25] Balduzzi S, Rücker G, Schwarzer G (2019). How to perform a meta-analysis with R: a practical tutorial. Evid Based Ment Health.

[26] Shen Y, Xu W, Li C, Handel A, Martinez L, Ling F, et al. A cluster of novel coronavirus disease 2019 infections indicating person-to-person transmission among casual contacts from social gatherings: an outbreak case-contact investigation. Open Forum Infect Dis. 2020;7(6):ofaa231–ofaa31. 10.1093/ofid/ofaa231 PMC7313868 32613025

[27] Song JS, Lee J, Kim M, Jeong HS, Kim MS, Kim SG (2022). Serial Intervals and Household Transmission of SARS-CoV-2 Omicron Variant, South Korea, 2021. Emerg Infect Dis.

[28] Manica M, De Bellis A, Guzzetta G, Mancuso P, Vicentini M, Venturelli F, et al. Intrinsic generation time of the SARS-CoV-2 Omicron variant: an observational study of household transmission. Lancet Reg Health Eur. 2022;19:100446. 10.1016/j.lanepe.2022.100446 PMC9246701 35791373

[29] Vazirinejad R, Khalili P, Jafarzadeh A, Shabani Z, Jamalizadeh A, Rezaei B, et al. A contact tracing prospective cohort retrieving epidemiological facts on SARS-CoV-2 transmission aspects; a serological analysis. Research Square. 2020.

[30] Mefsin YM, Chen D, Bond HS, Lin Y, Cheung JK, Wong JY, et al. Epidemiology of Infections with SARS-CoV-2 Omicron BA.2 Variant, Hong Kong, January-March 2022. Emerg Infect Dis. 2022;28(9):1856–8. 10.3201/eid2809.220613 PMC9423929 35914518

[31] Li L, Han ZG, Qin PZ, Liu WH, Yang Z, Chen ZQ, et al. Transmission and containment of the SARS-CoV-2 Delta variant of concern in Guangzhou, China: a population-based study. PLoS Negl Trop Dis. 2022;16(1):e0010048. 10.1371/journal.pntd.0010048 PMC8730460 34986169

[32] Bender JK, Brandl M, Höhle M, Buchholz U, Zeitlmann N (2021). Analysis of asymptomatic and presymptomatic transmission in SARS-CoV-2 outbreak, Germany, 2020. Emerg Infect Dis.

[33] Ma X, Wu K, Li Y, Li S, Cao L, Xie H (2022). Contact tracing period and epidemiological characteristics of an outbreak of the SARS-CoV-2 Delta variant in Guangzhou. Int J Infect Dis.

[34] Liu JY, Chen TJ, Hwang SJ (2020). Analysis of community-acquired COVID-19 cases in Taiwan. J Chin Med Assoc.

[35] Del Águila-Mejía J, Wallmann R, Calvo-Montes J, Rodríguez-Lozano J, Valle-Madrazo T, Aginagalde-Llorente A (2022). Secondary attack rate, transmission and incubation periods, and serial interval of SARS-CoV-2 Omicron variant, Spain. Emerg Infect Dis.

[36] Homma Y, Katsuta T, Oka H, Inoue K, Toyoshima C, Iwaki H, et al. The incubation period of the SARS-CoV-2 B1.1.7 variant is shorter than that of other strains. J Infect. 2021;83(2):e15–7. 10.1016/j.jinf.2021.06.011 PMC8225994 34146596

[37] Haddad N, Clapham HE, Abou Naja H, Saleh M, Farah Z, Ghosn N (2021). Calculating the serial interval of SARS-CoV-2 in Lebanon using 2020 contact-tracing data. BMC Infect Dis.

[38] Shi P, Gao Y, Shen Y, Chen E, Chen H, Liu J, et al. Characteristics and evaluation of the effectiveness of monitoring and control measures for the first 69 Patients with COVID-19 from 18 January 2020 to 2 March in Wuxi, China. Sustain Cities Soc. 2021;64:102559. 10.1016/j.scs.2020.102559 PMC7568471 33101882

[39] Kang M, Xin H, Yuan J, Taslim Ali S, Liang Z, Zhang J, et al. Transmission dynamics and epidemiological characteristics of SARS-CoV-2 Delta variant infections in Guangdong, China, May to June 2021. Euro Surveill. 2022;27(10):2100815. 10.2807/1560-7917.ES.2022.27.10.2100815 PMC8915401 35272744

[40] Ogata T, Tanaka H, Irie F, Hirayama A, Takahashi Y (2022). Shorter incubation period among unvaccinated Delta variant Coronavirus Disease 2019 patients in Japan. Int J Environ Res Public Health.

[41] Zhang L, Zhu J, Wang X, Yang J, Liu XF, Xu X-K. Characterizing COVID-19 transmission: incubation period, reproduction rate, and multiple-generation spreading. Front Phys. 2021;8:589963.

[42] Song R, Han B, Song M, Wang L, Conlon CP, Dong T, et al. Clinical and epidemiological features of COVID-19 family clusters in Beijing, China. J Infect. 2020;81(2):e26–30. 10.1016/j.jinf.2020.04.018 PMC7177072 32335171

[43] Brandal LT, MacDonald E, Veneti L, Ravlo T, Lange H, Naseer U, et al. Outbreak caused by the SARS-CoV-2 Omicron variant in Norway, November to December 2021. Euro Surveill. 2021;26(50):2101147. 10.2807/1560-7917.ES.2021.26.50.2101147 PMC8728491 34915975

[44] Geismar C, Fragaszy E, Nguyen V, Fong WLE, Shrotri M, Beale S, et al. Serial interval of COVID-19 and the effect of Variant B.1.1.7: analyses from prospective community cohort study (Virus Watch). Wellcome Open Res. 2021;6:224–4. 10.12688/wellcomeopenres.16974.1 PMC8564743 34796276

[45] Ryu S, Kim D, Lim JS, Ali ST, Cowling BJ. Serial interval and transmission dynamics during SARSCoV-2 Delta variant predominance, South Korea. Emerg Infect Dis. 2022;28(2):407–10. 10.3201/eid2802.211774 PMC8798673 34906289

[46] Kremer C, Braeye T, Proesmans K, André E, Torneri A, Hens N (2022). Serial Intervals for SARS-CoV-2 Omicron and Delta Variants, Belgium, November 19-December 31, 2021. Emerg Infect Dis.

[47] Backer JA, Eggink D, Andeweg SP, Veldhuijzen IK, van Maarseveen N, Vermaas K, et al. Shorter serial intervals in SARS-CoV-2 cases with Omicron BA.1 variant compared with Delta variant, the Netherlands, 13 to 26 December 2021. Euro Surveill. 2022;27(6):2200042. 10.2807/1560-7917.ES.2022.27.6.2200042 PMC8832521 35144721

[48] Kim D, Ali ST, Kim S, Jo J, Lim JS, Lee S (2022). Estimation of serial interval and reproduction number to quantify the transmissibility of SARS-CoV-2 Omicron variant in South Korea. Viruses.

[49] Gupta M, Parameswaran GG, Sra MS, Mohanta R, Patel D, Gupta A, et al. Contact tracing of COVID-19 in Karnataka, India: Superspreading and determinants of infectiousness and symptomatic infection. PLoS ONE. 2022;17(7):e0270789. 10.1371/journal.pone.0270789 PMC9273085 35816497

[50] Hwang H, Lim JS, Song SA, Achangwa C, Sim W, Kim G, et al. Transmission dynamics of the Delta variant of SARS-CoV-2 infections in South Korea. J Infect Dis. 2022;225(5):793–9. 10.1093/infdis/jiab586 34865022

[51] Li D, Li AE, Li ZQ, Bao Y, Liu T, Qin XR, et al. SARS-CoV-2 Delta variant in Jingmen City, Hubei Province, China, 2021: children susceptible and vaccination breakthrough infection. Front Microbiol. 2022;13:856757. 10.3389/fmicb.2022.856757 PMC9043846 35495649

[52] Tanaka H, Ogata T, Shibata T, Nagai H, Takahashi Y, Kinoshita M, et al. Shorter incubation period among COVID-19 vases with the BA.1 Omicron variant. Int J Environ Res Public Health. 2022;19(10):1–7. 10.3390/ijerph19106330 PMC9140418 35627870

[53] Bao C, Pan E, Ai J, Dai Q, Xu K, Shi N, et al. COVID-19 outbreak following a single patient exposure at an entertainment site: an epidemiological study. Transbound Emerg Dis. 2021;68(2):773–81. 10.1111/tbed.13742 32725765

[54] Shim E, Choi W, Kwon D, Kim T, Song Y. Transmission potential of the Omicron variant of Severe Acute Respiratory Syndrome Coronavirus 2 in South Korea, 25 November 2021-8 January 2022. Open Forum Infect Dis. 2022;9(7):ofac248. 10.1093/ofid/ofac248 PMC9129224 35855956

[55] Amato L, Candeloro L, Di Girolamo A, Savini L, Puglia I, Marcacci M, et al. Epidemiological and genomic findings of the first documented Italian outbreak of SARS-CoV-2 Alpha variant of concern. Epidemics. 2022;39:100578. 10.1016/j.epidem.2022.100578 PMC9098518 35636310

[56] Du Z, Xu X, Wu Y, Wang L, Cowling BJ, Meyers LA. Serial interval of COVID-19 among publicly reported confirmed cases. Emerg Infect Dis. 2020;26(6):1341–3. 10.3201/eid2606.200357 PMC7258488 32191173

[57] Song Y, Shim E (2022). Proportion of pre-symptomatic transmission events associated with COVID-19 in South Korea. J Clin Med.

[58] McAleavey P, Rainey E, McKaig C, Richardson C, Anderson C, Tilley C (2022). Outbreak of SARS-CoV-2 in a teenage discotheque in Northern Ireland-November 2021. Public Health.

[59] Sang Woo Park KS. Inferring the differences in incubation-period and generation-interval distributions of the Delta and Omicron variants of SARS-CoV-2. medRxiv. 2022. 10.1073/pnas.2221887120 PMC10235974 37216529

[60] Zhu W, Zhang M, Pan J, Yao Y, Wang W (2021). Effects of prolonged incubation period and centralized quarantine on the COVID-19 outbreak in Shijiazhuang, China: a modeling study. BMC Med.

[61] Allen H, Tessier E, Turner C, Anderson C, Blomquist P, Simons D, et al. Comparative transmission of SARS-CoV-2 Omicron (B.1.1.529) and Delta (B.1.617.2) variants and the impact of vaccination: national cohort study, England. medRxiv. 2022. 10.1017/S0950268823000420 PMC10125873 36938806

[62] Helmsdal G, Hansen OK, Møller LF, Christiansen DH, Petersen MS, Kristiansen MF (2022). Omicron outbreak at a private gathering in the Faroe Islands, infecting 21 of 33 triple vaccinated healthcare workers. Clin Infect Dis.

[63] Zhang M, Xiao J, Deng A, Zhang Y, Zhuang Y, Hu T, et al. Transmission dynamics of an outbreak of the COVID-19 Delta variant B.1.617.2 - Guangdong Province, China, May-June 2021. China CDC Wkly. 2021;3(27):584–6. 10.46234/ccdcw2021.148 PMC8392962 34594941

[64] Ping K, Lei M, Gou Y, Tao Z, Yao G, Hu C (2021). Epidemiologic characteristics of covid-19 in guizhou province, China. J Infect Dev Ctries.

[65] Liu Y, Zhao S, Ryu S, Ran J, Fan J, He D. Estimating the incubation period of SARS-CoV-2 Omicron BA.1 variant in comparison with that during the Delta variant dominance in South Korea. One Health. 2022;15:100425. 10.1016/j.onehlt.2022.100425 PMC9349028 35942477

[66] Deng B, Liu W, Guo Z, Luo L, Yang T, Huang J (2022). Natural history and cycle threshold values analysis of COVID-19 in Xiamen City, China. Infect Dis Model.

[67] Ki M. Epidemiologic characteristics of early cases with 2019 novel coronavirus (2019-nCoV) disease in Korea. Epidemiol Health. 2020;42:e2020007. 10.4178/epih.e2020007 PMC7285424 32035431

[68] Buchholz U, An Der Heiden M (2022). Serial interval in households infected with SARS-CoV-2 variant B.1.1.529 (Omicron) are even shorter compared to Delta. Epidemiol Infect..

[69] The SARS-CoV-2 variant with lineage B.1.351 clusters investigation team. Linked transmission chains of imported SARS-CoV-2 variant B.1.351 across mainland France, January 2021. Euro Surveill. 2021;26(13):2100333. 10.2807/1560-7917.ES.2021.26.13.2100333 PMC8017907 33797392

[70] Mao S, Huang T, Yuan H, Li M, Huang X, Yang C (2020). Epidemiological analysis of 67 local COVID-19 clusters in Sichuan Province, China. BMC Public Health.

[71] Ali ST, Chen D, Lim WW, Yeung A, Adam DC, Lau YC, et al. Insights into COVID-19 epidemiology and control from temporal changes in serial interval distributions in Hong Kong. medRxiv. 2022. 10.1093/aje/kwae220 39013785

[72] Yan X, Xiao W, Zhou S, Wang X, Wang Z, Zhao M (2022). A four-generation family transmission chain of COVID-19 along the China-Myanmar border in October to November 2021. Front Public Health.

[73] Zhang X, Wang H, Wang Y, Lei Y, Xu K, Zhang J (2020). Epidemiological and clinical based study on four passages of COVID-19 patients: intervention at asymptomatic period contributes to early recovery. BMC Infect Dis.

[74] Xiong W, Peng L, Tsang TK, Cowling BJ (2023). Epidemiology of SARS-CoV-2 Omicron BA.5 Infections, Macau, June-July 2022. Emerg Infect Dis.

[75] Wright E, Pollard G, Robertson H, Anuradha S. Household transmission of the Delta COVID-19 variant in Queensland, Australia: a case series. Epidemiol Infect. 2022;150:e173–e73. 10.1017/S0950268822001546 PMC9671917 36192365

[76] Wei Z, Ma W, Wang Z, Li J, Fu X, Chang H, et al. Household transmission of SARS-CoV-2 during the Omicron wave in Shanghai, China: a case-ascertained study. Influenza Other Respi Viruses. 2023;17(2):e13097. 10.1111/irv.13097 PMC9946695 36843225

[77] Yu X, Sun X, Cui P, Pan H, Lin S, Han R (2020). Epidemiological and clinical characteristics of 333 confirmed cases with coronavirus disease 2019 in Shanghai, China. Transbound Emerg Dis.

[78] Wang K, Guo Z, Zeng T, Sun S, Lu Y, Wang J, et al. Transmission characteristics and inactivated vaccine effectiveness against transmission of SARS-CoV-2 Omicron BA.5 variants in Urumqi, China. JAMA Netw Open. 2023;6(3):e235755–e55. 10.1001/jamanetworkopen.2023.5755 PMC10064257 36995713

[79] Wang J, Ma T, Ding S, Xu K, Zhang M, Zhang Z, et al. Dynamic characteristics of a COVID-19 outbreak in Nanjing, Jiangsu province, China. Front Public Health. 2022;10:933075. 10.3389/fpubh.2022.933075 PMC9723226 36483256

[80] Ogata T, Tanaka H. SARS-CoV-2 incubation period during the Omicron BA.5- dominant period in Japan. Emerg Infect Dis. 2023;29(3):595–8. 10.3201/eid2903.221360 PMC9973685 36787734

[81] Luo K, Wu Y, Wang Y, Liu Z, Yi L, Zhao S, et al. Transmission dynamics and epidemiological characteristics of the SARS-CoV-2 Delta variant - Hunan Province, China, 2021. China CDC Wkly. 2023;5(3):56–62. 10.46234/ccdcw2023.011 PMC9902755 36776461

[82] Guo Z, Zhao S, Yam CHK, Li C, Jiang X, Chow TY, et al. Estimating the serial intervals of SARS-CoV-2 Omicron BA.4, BA.5, and BA.2.12.1 variants in Hong Kong. Influenza Other Respi Viruses. 2023;17(2):e13105. 10.1111/irv.13105 PMC9942273 36824395

[83] Guo Z, Zhao S, Mok CKP, So RTY, Yam CHK, Chow TY, et al. Comparing the incubation period, serial interval, and infectiousness profile between SARS-CoV-2 Omicron and Delta variants. J Med Virol. 2023;95(3):e28648. 10.1002/jmv.28648 36892159

[84] Geismar C, Nguyen V, Fragaszy E, Shrotri M, Navaratnam AMD, Beale S (2022). Bayesian reconstruction of household transmissions to infer the serial interval of COVID-19 by variants of concern: analysis from a prospective community cohort study (Virus Watch). Lancet.

[85] Guo CX, He L, Yin JY, Meng XG, Tan W, Yang GP (2020). Epidemiological and clinical features of pediatric COVID-19. BMC Med.

[86] Nie X, Fan L, Mu G, Tan Q, Wang M, Xie Y (2020). Epidemiological characteristics and incubation period of 7015 confirmed cases with Coronavirus Disease 2019 outside Hubei Province in China. J Infect Dis.

[87] Yu S, Cui S, Rui J, Zhao Z, Deng B, Liu C, et al. Epidemiological characteristics and transmissibility for SARS-CoV-2 of population level and cluster level in a Chinese City. Front Public Health. 2021;9:799536. 10.3389/fpubh.2021.799536 PMC8805998 35118044

[88] Wang P, Lu JA, Jin Y, Zhu M, Wang L, Chen S (2020). Statistical and network analysis of 1212 COVID-19 patients in Henan, China. Int J Infect Dis..

[89] Son H, Lee H, Lee M, Eun Y, Park K, Kim S, et al. Epidemiological characteristics of and containment measures for COVID-19 in Busan, Korea. Epidemiol Health. 2020;42:e2020035. 10.4178/epih.e2020035 PMC7644939 32512664

[90] Baskaradoss JK, Alsumait A, Malik S, Ariga J, Geevarghese A, Francis R (2021). Epidemiological characteristics of COVID-19 cases among indians residing in Kuwait. East Mediterr Health J.

[91] Bae S, Kim H, Jung TY, Lim JA, Jo DH, Kang GS, et al. Epidemiological characteristics of COVID-19 outbreak at fitness centers in Cheonan, Korea. J Korean Med Sci. 2020;35(31):e288. 10.3346/jkms.2020.35.e288 PMC7416003 32776726

[92] Du S, Lu H, Su Y, Wang X, Bi S, Wu J (2021). Epidemiological characteristics of COVID-19 under government-mandated control measures during January-February 2020 in Inner Mongolia, China. Jpn J Infect Dis.

[93] Thway AM, Teza H, Win TT, Htun YM, Aung MM, Win YN, et al. Epidemiological characteristics of SARS-COV-2 in Myanmar. medRxiv. 2020.

[94] Kwok KO, Wong VWY, Wei WI, Wong SYS, Tang JWT. Epidemiological characteristics of the first 53 laboratory-confirmed cases of COVID-19 epidemic in Hong Kong, 13 February 2020. Euro Surveill. 2020;25(16):2000155. 10.2807/1560-7917.ES.2020.25.16.2000155 PMC7189647 32347198

[95] Lai CKC, Ng RWY, Wong MCS, Chong KC, Yeoh YK, Chen Z (2020). Epidemiological characteristics of the first 100 cases of coronavirus disease 2019 (COVID-19) in Hong Kong Special Administrative Region, China, a city with a stringent containment policy. Int J Epidemiol.

[96] Jia J, Hu X, Yang F, Song X, Dong L, Zhang J, et al. Epidemiological characteristics on the clustering nature of COVID-19 in Qingdao City, 2020: a descriptive analysis. Disaster Med Public Health Prep. 2020;14(5):643–7. 10.1017/dmp.2020.59 PMC7156571 32228732

[97] Hua CZ, Miao ZP, Zheng JS, Huang Q, Sun QF, Lu HP (2020). Epidemiological features and viral shedding in children with SARS-CoV-2 infection. J Med Virol.

[98] Wong J, Chaw L, Koh WC, Alikhan MF, Jamaludin SA, Poh WWP, et al. Epidemiological investigation of the first 135 COVID-19 cases in Brunei: implications for surveillance, control, and travel restrictions. Am J Trop Med Hyg. 2020;103(4):1608–13. 10.4269/ajtmh.20-0771 PMC7543844 32815514

[99] Haw NJL, Uy J, Sy KTL, Abrigo MRM. Epidemiological profile and transmission dynamics of COVID-19 in the Philippines. Epidemiol Infect. 2020;148:e204. 10.1017/S0950268820002137 PMC7506175 32928322

[100] Zhang Q, Zhu J, Jia C, Xu S, Jiang T, Wang S. Epidemiology and clinical outcomes of COVID-19 patients in Northwestern China who had a history of exposure in Wuhan City: departure time-originated pinpoint surveillance. Front Med (Lausanne). 2021;8:582299. 10.3389/fmed.2021.582299 PMC8192719 34124080

[101] Ganyani T, Kremer C, Chen D, Torneri A, Faes C, Wallinga J, et al. Estimating the generation interval for coronavirus disease (COVID-19) based on symptom onset data, March 2020. Euro Surveill. 2020;25(17):2000257. 10.2807/1560-7917.ES.2020.25.17.2000257 PMC7201952 32372755

[102] Hart WS, Abbott S, Endo A, Hellewell J, Miller E, Andrews N, et al. Inference of the SARS-CoV-2 generation time using UK household data. Elife. 2022;11:e70767. 10.7554/eLife.70767 PMC8967386 35138250

[103] Zhao S, Tang B, Musa SS, Ma S, Zhang J, Zeng M, et al. Estimating the generation interval and inferring the latent period of COVID-19 from the contact tracing data. Epidemics. 2021;36:100482. 10.1016/j.epidem.2021.100482 PMC8223005 34175549

[104] Lau YC, Tsang TK, Kennedy-Shaffer L, Kahn R, Lau EHY, Chen D, et al. Joint estimation of generation time and incubation period for Coronavirus Disease (Covid-19). J Infect Dis. 2021;224(10):1664–71. 10.1093/infdis/jiab424 PMC8499762 34423821

[105] Deng Y, You C, Liu Y, Qin J, Zhou XH (2021). Estimation of incubation period and generation time based on observed length-biased epidemic cohort with censoring for COVID-19 outbreak in China. Biometrics.

[106] Böhm S, Woudenberg T, Chen D, Marosevic DV, Böhmer MM, Hansen L, et al. Epidemiology and transmission characteristics of early COVID-19 cases, 20 January-19 March 2020, in Bavaria, Germany. Epidemiol Infect. 2021;149:e65. 10.1017/S0950268821000510 PMC7985897 33650470

[107] Böhmer MM, Buchholz U, Corman VM, Hoch M, Katz K, Marosevic DV (2020). Investigation of a COVID-19 outbreak in Germany resulting from a single travel-associated primary case: a case series. Lancet Infect Dis.

[108] Wu J, Huang Y, Tu C, Bi C, Chen Z, Luo L, et al. Household transmission of SARS-CoV-2, Zhuhai, China, 2020. Clin Infect Dis. 2020;71(16):2099–108. 10.1093/cid/ciaa557 PMC7239243 32392331

[109] Yang L, Dai J, Zhao J, Wang Y, Deng P, Wang J. Estimation of incubation period and serial interval of COVID-19: analysis of 178 cases and 131 transmission chains in Hubei province, China. Epidemiol Infect. 2020;148:e117. 10.1017/S0950268820001338 PMC7324649 32594928

[110] Zhang J, Litvinova M, Wang W, Wang Y, Deng X, Chen X (2020). Evolving epidemiology and transmission dynamics of coronavirus disease 2019 outside Hubei province, China: a descriptive and modelling study. Lancet Infect Dis.

[111] Backer JA, Klinkenberg D, Wallinga J. Incubation period of 2019 novel coronavirus (2019-nCoV) infections among travellers from Wuhan, China, 20 28 January 2020. Euro Surveill. 2020;25(5):2000062. 10.2807/1560-7917.ES.2020.25.5.2000062 PMC7014672 32046819

[112] Bui LV, Nguyen HT, Levine H, Nguyen HN, Nguyen TA, Nguyen TP, et al. Estimation of the incubation period of COVID-19 in Vietnam. PLoS ONE. 2020;15(12):e0243889. 10.1371/journal.pone.0243889 PMC7757798 33362233

[113] Cheng C, Zhang D, Dang D, Geng J, Zhu P, Yuan M, et al. Incubation period of COVID-19 from 11545 patients in observation study. Research Square. 2021. 10.1186/s40249-021-00901-9 PMC8446477 34535192

[114] Han T. Outbreak investigation: transmission of COVID-19 started from a spa facility in a local community in Korea. Epidemiol Health. 2020;42:e2020056. 10.4178/epih.e2020056 PMC7871164 32777883

[115] Kong TK (2020). Longer incubation period of Coronavirus Disease 2019 (COVID-19) in older adults. Aging Med (Milton).

[116] Men K, Wang X, Yihao L, Zhang G, Hu J, Gao Y, et al. Estimate the incubation period of coronavirus 2019 (COVID-19). medRxiv. 2020. 10.1016/j.compbiomed.2023.106794 PMC10062796 37044045

[117] Xiao Z, Guo W, Luo Z, Liao J, Wen F, Lin Y (2021). Examining geographical disparities in the incubation period of the COVID-19 infected cases in Shenzhen and Hefei, China. Environ Health Prev Med.

[118] Xiao Z, Xie X, Guo W, Luo Z, Liao J, Wen F (2020). Examining the incubation period distributions of COVID-19 on Chinese patients with different travel histories. J Infect Dev Ctries.

[119] Ratovoson R, Razafimahatratra R, Randriamanantsoa L, Raberahona M, Rabarison HJ, Rahaingovahoaka FN, et al. Household transmission of COVID-19 among the earliest cases in Antananarivo, Madagascar. Influenza Other Respi Viruses. 2022;16(1):48–55. 10.1111/irv.12896 PMC8447323 34378341

[120] Aghaali M, Kolifarhood G, Nikbakht R, Saadati HM, Hashemi Nazari SS. Estimation of the serial interval and basic reproduction number of COVID-19 in Qom, Iran, and three other countries: a data-driven analysis in the early phase of the outbreak. Transbound Emerg Dis. 2020;67(6):2860–8. 10.1111/tbed.13656 PMC7300937 32473049

[121] Bi Q, Wu Y, Mei S, Ye C, Zou X, Zhang Z (2020). Epidemiology and transmission of COVID-19 in 391 cases and 1286 of their close contacts in Shenzhen, China: a retrospective cohort study. Lancet Infect Dis.

[122] Chu VT, Yousaf AR, Chang K, Schwartz NG, McDaniel CJ, Lee SH (2021). Household Transmission of SARS-CoV-2 from Children and Adolescents. N Engl J Med.

[123] Expert Taskforce for the C-CSO. Epidemiology of COVID-19 outbreak on cruise ship quarantined at Yokohama, Japan, February 2020. Emerg Infect Dis. 2020;26(11):2591–7. 10.3201/eid2611.201165 PMC7588545 32822290

[124] McAloon CG, Wall P, Griffin J, Casey M, Barber A, Codd M (2021). Estimation of the serial interval and proportion of pre-symptomatic transmission events of COVID- 19 in Ireland using contact tracing data. BMC Public Health.

[125] Ng SH-X, Kaur P, Kremer C, Tan WS, Tan AL, Hens N, et al. Estimating transmission parameters for COVID-19 clusters by using symptom onset data, Singapore, January-April 2020. Emerg Infect Dis. 2021;27(2):582–5. 10.3201/eid2702.203018 PMC7853533 33496243

[126] Talmoudi K, Safer M, Letaief H, Hchaichi A, Harizi C, Dhaouadi S (2020). Estimating transmission dynamics and serial interval of the first wave of COVID-19 infections under different control measures: a statistical analysis in Tunisia from February 29 to May 5, 2020. BMC Infect Dis.

[127] Wang K, Zhao S, Liao Y, Zhao T, Wang X, Zhang X (2020). Estimating the serial interval of the novel coronavirus disease (COVID-19) based on the public surveillance data in Shenzhen, China, from 19 January to 22 February 2020. Transbound Emerg Dis.

[128] Kwok KO, Wei WI, Huang Y, Kam KM, Chan EYY, Riley S, et al. Evolving epidemiological characteristics of COVID-19 in Hong Kong from January to August 2020: retrospective study. J Med Internet Res. 2021;23(4):e26645. 10.2196/26645 PMC8054773 33750740

[129] Chong DWQ, Jayaraj VJ, Ng CW, Sam IC, Said MA, Ahmad Zaki R (2021). Propagation of a hospitalassociated cluster of COVID-19 in Malaysia. BMC Infect Dis.

[130] Dai J, Yang L, Zhao J (2020). Probable longer incubation period for elderly Covid-19 cases: analysis of 180 contact tracing data in Hubei province, China. Risk Manag Healthc Policy.

[131] Huang L, Zhang X, Zhang X, Wei Z, Zhang L, Xu J, et al. Rapid asymptomatic transmission of COVID-19 during the incubation period demonstrating strong infectivity in a cluster of youngsters aged 16-23 years outside Wuhan and characteristics of young patients with COVID-19: a prospective contact-tracing study. J Infect. 2020;80(6):e1–3. 10.1016/j.jinf.2020.03.006 PMC7194554 32283156

[132] Li M, Chen P, Yuan Q, Song B, Ma J. Transmission characteristics of the COVID-19 outbreak in China: a study driven by data. medRxiv. 2020.

[133] Areekal B, Vijayan SM, Suseela MS, Andrews MA, Ravi RK, Sukumaran ST, et al. Risk factors, epidemiological and clinical outcome of close contacts of Covid-19 cases in a tertiary hospital in Southern India. J Clin Diagnostic Res. 2021;15(3):LC34–7.

[134] Cao Y, Wang Y, Das A, Pan CQ, Xie W. Transmission dynamics of COVID-19 among index case family clusters in Beijing, China. Epidemiol Infect. 2020;149:e74. 10.1017/S0950268820002848 PMC8007952 33208219

[135] Chun JY, Baek G, Kim Y (2020). Transmission onset distribution of COVID-19. Int J Infect Dis.

[136] Tindale LC, Stockdale JE, Coombe M, Garlock ES, Lau WYV, Saraswat M, et al. Evidence for transmission of COVID-19 prior to symptom onset. Elife. 2020;9:e57149. 10.7554/eLife.57149 PMC7386904 32568070

[137] Won YS, Kim JH, Ahn CY, Lee H. Subcritical transmission in the early stage of Covid-19 in Korea. Int J Environ Res Public Health. 2021;18(3):1–10. 10.3390/ijerph18031265 PMC7908312 33572542

[138] Xia W, Liao J, Li C, Li Y, Qian X, Sun X, et al. Transmission of Corona Virus Disease 2019 during the incubation period may lead to a quarantine loophole. medRxiv. 2020.

[139] Zhao H, Zhang Z, Lun W, Chen Z, Lu X, Li J, et al. Transmission dynamics and successful control measures of SARS-CoV-2 in the mega-size city of Guangzhou, China. Medicine (Baltimore). 2021;100(48):e27846. 10.1097/MD.0000000000027846 PMC9191374 35049185

[140] Lauer SA, Grantz KH, Bi Q, Jones FK, Zheng Q, Meredith HR, et al. The incubation period of Coronavirus Disease 2019 (CoVID-19) from publicly reported confirmed cases: estimation and application. Ann Intern Med. 2020;172(9):577–82. 10.7326/M20-0504 PMC7081172 32150748

[141] Emecen AN, Basoglu Sensoy E, Sezgin E, Yildirim Ustuner B, Keskin S, Siyve N (2021). Transmission dynamics and timing of key events for SARS-CoV-2 infection in healthcare workers. Infect Dis (Lond).

[142] Li M, Liu K, Song Y, Wang M, Wu J. Serial interval and generation interval for imported and local infectors, respectively, estimated using reported contact-tracing data of COVID-19 in China. Front Public Health. 2020;8:577431. 10.3389/fpubh.2020.577431 PMC7821042 33490015

[143] Bernal Lopez J, Panagiotopoulos N, Byers C, Vilaplana TG, Boddington N, Zhang XS, et al. Transmission dynamics of COVID-19 in household and community settings in the United Kingdom, January to March 2020. Euro Surveill. 2022;27(15):2001551. 10.2807/1560-7917.ES.2022.27.15.2001551 PMC9012093 35426357

[144] Cereda D, Manica M, Tirani M, Rovida F, Demicheli V, Ajelli M, et al. The early phase of the COVID-19 epidemic in Lombardy, Italy. Epidemics. 2021;37:100528. 10.1016/j.epidem.2021.100528 PMC8605863 34814093

[145] Hong K, Yum S, Kim J, Chun BC. The serial interval of COVID-19 in Korea: 1,567 pairs of symptomatic cases from contact tracing. J Korean Med Sci. 2020;35(50):e435. 10.3346/jkms.2020.35.e435 PMC7769696 33372427

[146] Lavezzo E, Franchin E, Ciavarella C, Cuomo-Dannenburg G, Barzon L, Del Vecchio C, et al. Suppression of a SARS-CoV-2 outbreak in the Italian municipality of Vo'. Nature. 2020;584(7821):425–9. 10.1038/s41586-020-2488-1 PMC7618354 32604404

[147] Liu T, Qi L, Yao M, Tian K, Lin M, Jiang H, et al. Serial interval and reproductive number of COVID-19 among 116 infector-infectee pairs - Jingzhou City, Hubei province, China, 2020. China CDC Wkly. 2020;2(27):491–5. 10.46234/ccdcw2020.118 PMC8428449 34594686

[148] Najafi F, Izadi N, Hashemi-Nazari SS, Khosravi-Shadmani F, Nikbakht R, Shakiba E. Serial interval and time-varying reproduction number estimation for COVID-19 in western Iran. New Microbes New Infect. 2020;36:100715. 10.1016/j.nmni.2020.100715 PMC7293842 32566233

[149] Prete CA, Buss L, Dighe A, Porto VB, da Silva Candido D, Ghilardi F, et al. Serial interval distribution of SARS-CoV-2 infection in Brazil. J Travel Med. 2021;28(2):taaa115. 10.1093/jtm/taaa115 PMC7454808 32710618

[150] Reed IG, Walker ES, Landguth EL. SARS-CoV-2 Serial Interval Variation, Montana, USA, March 1-July 31, 2020. Emerg Infect Dis. 2021;27(5):1486–91. 10.3201/eid2705.204663 PMC8084495 33900189

[151] Ryu S, Ali ST, Noh E, Kim D, Lau EHY, Cowling BJ. Transmission dynamics and control of two epidemic waves of SARS-CoV-2 in South Korea. BMC Infect Dis. 2021;21(1):1–9. 10.1186/s12879-021-06204-6 PMC8154110 34039296

[152] Saurabh S, Verma MK, Gautam V, Kumar N, Goel AD, Gupta MK, et al. Transmission dynamics of the COVID-19 epidemic at the district level in India: prospective observational study. JMIR Public Health Surveill. 2020;6(4):e22678. 10.2196/22678 PMC7572116 33001839

[153] Thai PQ, Rabaa MA, Luong DH, Tan DQ, Quang TD, Quach HL, et al. The first 100 days of Severe Acute Respiratory Syndrome Coronavirus 2 (SARS-CoV-2) control in Vietnam. Clin Infect Dis. 2021;72(9):E334–E42. 10.1093/cid/ciaa1130 PMC7454342 32738143

[154] Wang Y, Siesel C, Chen Y, Lopman B, Edison L, Thomas M, et al. Severe Acute Respiratory Syndrome Coronavirus 2 transmission in Georgia, USA, February 1-July 13, 2020. Emerg Infect Dis. 2021;27(10):2578–87. 10.3201/eid2710.210061 PMC8462336 34399085

[155] Qin J, You C, Lin Q, Hu T, Yu S, Zhou XH. Estimation of incubation period distribution of COVID-19 using disease onset forward time: A novel cross-sectional and forward follow-up study. Sci Adv. 2020;6(33):eabc1202. 10.1126/sciadv.abc1202 PMC7428324 32851189

[156] Ren X, Li Y, Yang X, Li Z, Cui J, Zhu A (2021). Evidence for pre-symptomatic transmission of Coronavirus Disease 2019 (COVID-19) in China. Influenza Other Respi Viruses.

[157] Zhao S, Gao D, Zhuang Z, Chong MKC, Cai Y, Ran J (2020). Estimating the serial interval of the Novel Coronavirus Disease (COVID-19): a statistical analysis using the public data in Hong Kong from January 16 to February 15, 2020. Front Phys.

[158] Adam DC, Wu P, Wong JY, Lau EHY, Tsang TK, Cauchemez S (2020). Clustering and superspreading potential of SARS-CoV-2 infections in Hong Kong. Nat Med.

[159] Ferretti L, Wymant C, Kendall M, Zhao L, Nurtay A, Abeler-Dorner L, et al. Quantifying SARS-CoV-2 transmission suggests epidemic control with digital contact tracing. Science. 2020;368(6491):eabb6936. 10.1126/science.abb6936 PMC7164555 32234805

[160] Althobaity Y, Wu J, Tildesley MJ (2022). A comparative analysis of epidemiological characteristics of MERS-CoV and SARS-CoV-2 in Saudi Arabia. Infect Dis Model.

[161] Wang Y, Sun K, Pan Y, Yi L, Huo D, Wu Y, et al. SARS-CoV-2 containment was achievable during the early stage of the pandemic: a retrospective modelling study of the Xinfadi outbreak in Beijing. medRxiv. 2022.

[162] Achangwa C, Park H, Ryu S. Incubation period of wild type of SARS-CoV-2 infections by age, gender, and epidemic periods. Front Public Health. 2022;10:905020. 10.3389/fpubh.2022.905020 PMC9363879 35968429

[163] Zhao Q, Zhang Y, Li M, Tian R, Zhao Y, Cao B, et al. Epidemiological clustered characteristics of Coronavirus Disease 2019 (COVID-19) in three phases of transmission in Jilin Province, China. PLoS ONE. 2023;18(1):e0279879. 10.1371/journal.pone.0279879 PMC9851530 36656818

[164] Somda SMA, Ouedraogo B, Pare CB, Kouanda S. Estimation of the serial interval and the effective reproductive number of COVID-19 outbreak using contact data in Burkina Faso, a Sub-Saharan African country. Comput Math Methods Med. 2022;2022:8239915. 10.1155/2022/8239915 PMC9527438 36199779

[165] Hammond V, Butchard M, Stablein H, Jack S (2022). COVID-19 in one region of New Zealand: a descriptive epidemiological study. Aust N Z J Public Health.

[166] Li Q, Guan X, Wu P, Wang X, Zhou L, Tong Y (2020). Early transmission dynamics in Wuhan, China, of novel coronavirus-infected pneumonia. N Engl J Med.

[167] Ma S, Zhang J, Zeng M, Yun Q, Guo W, Zheng Y, et al. Epidemiological parameters of Coronavirus Disease 2019: a pooled analysis of publicly reported individual data of 1155 cases from seven countries. medRxiv. 2020.

[168] Rathore V, Galhotra A, Pal R, Sahu KK (2020). COVID-19 pandemic and children: a review. J Pediatr Pharmacol Ther.

[169] Zimmermann P, Curtis N (2020). COVID-19 in children, pregnancy and neonates: a review of epidemiologic and clinical features. Pediatr Infect Dis J.

[170] Daley C, Fydenkevez M, Ackerman-Morris S. A systematic review of the incubation period of SARS-CoV-2: the effects of age, biological sex, and location on incubation period. medRxiv. 2020.

[171] Li Z, Zhang Y, Wang C, Peng L, Gao R, Jing J, et al. The incubation period of Severe Acute Respiratory Syndrome Coronavirus 2: a systematic review. medRxiv. 2020.

[172] Wang Y, Cao Z, Zeng DD, Zhang Q, Luo T (2021). The collective wisdom in the COVID-19 research: comparison and synthesis of epidemiological parameter estimates in preprints and peer reviewed articles. Int J Infect Dis.

[173] Wei Y, Wei L, Liu Y, Huang L, Shen S, Zhang R (2021). Comprehensive estimation for the length and dispersion of COVID-19 incubation period: a systematic review and meta-analysis. Infection.

[174] Xie Y, Wang Z, Liao H, Marley G, Wu D, Tang W (2020). Epidemiologic, clinical, and laboratory findings of the COVID-19 in the current pandemic: systematic review and meta-analysis. BMC Infect Dis.

[175] Zaki N, Mohamed EA (2021). The estimations of the COVID-19 incubation period: a scoping reviews of the literature. J Infect Public Health.

[176] Song WL, Zou N, Guan WH, Pan JL, Xu W (2021). Clinical characteristics of COVID-19 in family clusters: a systematic review. World J Pediatr.

[177] Griffin J, Casey M, Collins Á, Hunt K, McEvoy D, Byrne A, et al. Rapid review of available evidence on the serial interval and generation time of COVID-19. BMJ Open. 2020;10(11):e040263. 10.1136/bmjopen-2020-040263 PMC7684810 33234640

[178] Allen K, Parry AE, Glass K (2021). Early reports of epidemiological parameters of the COVID-19 pandemic. Western Pac Surveill Response J.

[179] Biggerstaff M, Cowling BJ, Cucunubá ZM, Dinh L, Ferguson NM, Gao H, et al. Early insights from statistical and mathematical modeling of key epidemiologic parameters of COVID-19. Emerg Infect Dis. 2020;26(11):E1–E14. 10.3201/eid2611.201074 PMC7588530 32917290

[180] Alene M, Yismaw L, Assemie MA, Ketema DB, Gietaneh W, Birhan TY (2021). Serial interval and incubation period of COVID-19: a systematic review and meta-analysis. BMC Infect Dis.

[181] Cheng C, Zhang DD, Dang D, Geng J, Zhu P, Yuan M (2021). The incubation period of COVID-19: a global meta-analysis of 53 studies and a Chinese observation study of 11 545 patients. Infect Dis Poverty.

[182] Dhouib W, Maatoug J, Ayouni I, Zammit N, Ghammem R, Fredj SB, et al. The incubation period during the pandemic of COVID-19: a systematic review and meta-analysis. Syst Rev. 2021;10(1):1–14. 10.1186/s13643-021-01648-y PMC8031340 33832511

[183] Elias C, Sekri A, Leblanc P, Cucherat M, Vanhems P. The incubation period of COVID-19: a meta-analysis. Int J Infect Dis. 2021;104:708–10. 10.1016/j.ijid.2021.01.069 PMC7857041 33548553

[184] Izadi N, Taherpour N, Mokhayeri Y, Sotoodeh Ghorbani S, Rahmani K, Hashemi Nazari SS. Epidemiologic parameters for COVID-19: a systematic review and meta-analysis. Med J Islam Repub Iran. 2022;36:155. 10.47176/mjiri.36.155 PMC9832936 36654849

[185] Khalili M, Karamouzian M, Nasiri N, Javadi S, Mirzazadeh A, Sharifi H. Epidemiological characteristics of COVID-19; a systemic review and meta-analysis. medRxiv. 2020. 10.1017/S0950268820001430 PMC7343974 32594937

[186] Li B, Zhang S, Zhang R, Chen X, Wang Y, Zhu C. Epidemiological and clinical characteristics of COVID-19 in children: a systematic review and meta-analysis. Front Pediatr. 2020;8:591132. 10.3389/fped.2020.591132 PMC7667131 33224909

[187] Quesada JA, López-Pineda A, Gil-Guillén VF, Arriero-Marín JM, Gutiérrez F, Carratala-Munuera C. Incubation period of COVID-19: a systematic review and meta-analysis. Rev Clin Esp. 2021;221(2):109–17. 10.1016/j.rceng.2020.08.002 PMC7698828 33998486

[188] Rai B, Shukla A, Dwivedi LK. Incubation period for COVID-19: a systematic review and meta-analysis. J Public Health. 2021:1–8. 10.1007/s10389-021-01478-1 PMC7901514 33643779

[189] Rai B, Shukla A, Dwivedi LK. Estimates of serial interval for COVID-19: a systematic review and meta-analysis. Clin Epidemiol Glob Health. 2021;9:157–61. 10.1016/j.cegh.2020.08.007 PMC7448781 32869006

[190] Rampal S, Jayaraj VJ, Chong DWQ, Ng C-W. A meta-analysis of the serial interval and generation time of COVID-19 transmission. Int J Epidemiol. 2021;50.

[191] Wassie GT, Azene AG, Bantie GM, Dessie G, Aragaw AM. Incubation period of severe acute respiratory syndrome novel Coronavirus 2 that causes Coronavirus disease 2019: a systematic review and meta-analysis. Curr Ther Res Clin Exp. 2020;93:100607. 10.1016/j.curtheres.2020.100607 PMC7548076 33071295

[192] Wei Y, Wei L, Liu Y, Huang L, Shen S, Zhang R, et al. A systematic review and meta-analysis reveals long and dispersive incubation period of COVID-19. medRxiv. 2020.

[193] Xin H, Wong JY, Murphy C, Yeung A, Taslim Ali S, Wu P, et al. The incubation period distribution of Coronavirus disease 2019: a systematic review and meta-analysis. Clin Infect Dis. 2021;73(12):2344–52. 10.1093/cid/ciab501 34117868

[194] Zhang T, Ding S, Zeng Z, Cheng H, Zhang C, Mao X, et al. Estimation of incubation period and serial interval for SARS-CoV-2 in Jiangxi, China, and an Updated Meta-Analysis. J Infect Dev Ctries. 2021;15(3):326–32. 10.3855/jidc.14025 33839705

[195] Yang Y, Kenah E. Understanding how fast SARS-CoV-2 variants transmit from household studies. Lancet Infect Dis. 2022;22(5):564–5. 10.1016/S1473-3099(22)00053-6 PMC8843065 35176229

[196] Lee M, Fayad M, Mabud T, De Lara PT, Hernandez AE, Contreras Anez GA, et al. Clinical characteristics of early noncritical hospitalized patients with coronavirus disease 2019 (COVID-19): a single-center retrospective study in New York City. Open Forum Infect Dis. 2020;7(SUPPL 1):S287–S88.

[197] Mersini K, Vasili A, Dervishi M, Kureta E, Robo A, Alla L, et al. Early phase of COVID-19 epidemic in Albania. Int J Infect Dis. 2022;116:S26–7.

[198] Sukhyun R, Achangwa C. No difference in the incubation period of COVID-19 in different gender, ages, and epidemic periods in South Korea. Int J Infect Dis. 2022;116:S53.

[199] Werajong O, Denduangchai S, Deepreecha K, Bhopdhornangkul B. The characteristic of the COVID-19 outbreak in The Royal Thai Army personal during 1st April to 8th May 2021 and Preventive Policy suggestion. Saf Health Work. 2022;13:S175.

[200] Wu J, Chen X, Gong L, Huo S, Gao X, Nie S, et al. Epidemiological and clinical features of SARS-CoV-2 cluster infection in Anhui Province, Eastern China. Int J Infect Dis. 2022;117:372–7. 10.1016/j.ijid.2021.04.064 PMC8110625 33984511

[201] Wang Y, Wang Q, Wang K, Song C, Guo Z, Hu W. A case of COVID-19 with an ultralong incubation period. Infect Control Hosp Epidemiol. 2021;42(2):242–3. 10.1017/ice.2020.221 PMC7298099 32389131

[202] Soedarsono S. A family cluster of Coronavirus disease (COVID-19) infection with different clinical manifestations. Acta Med Indones. 2020;52(2):155–62. 32778630

[203] Du X, Kang K, Chong Y, Zhang ML, Yang W, Meng XL, et al. COVID-19 patient with an incubation period of 27 d: a case report. World J Clin Cases. 2021;9(21):5955–62. 10.12998/wjcc.v9.i21.5955 PMC8316961 34368314

[204] Koff AG, Laurent-Rolle M, Hsu JCC, Malinis M. Prolonged incubation of severe acute respiratory syndrome coronavirus 2 (SARS-CoV-2) in a patient on rituximab therapy. Infect Control Hosp Epidemiol. 2021;42(10):1286–8. 10.1017/ice.2020.1239 PMC7578652 33023685

[205] Zheng Y, Xiong C, Liu Y, Qian X, Tang Y, Liu L, et al. Epidemiological and clinical characteristics analysis of COVID-19 in the surrounding areas of Wuhan, Hubei Province in 2020. Pharmacol Res. 2020;157:104821. 10.1016/j.phrs.2020.104821 PMC7191275 32360481

[206] Ito K, Piantham C, Nishiura H. Estimating relative generation times and reproduction numbers of Omicron BA.1 and BA.2 with respect to Delta variant in Denmark. Math Biosci Eng. 2022;19(9):9005–17. 10.3934/mbe.2022418 35942746

[207] Capon A, Ousta D, Ferson M, Ingleton A, Sheppeard V. A multiple site community outbreak of COVID-19 in Sydney, Australia. Aust N Z J Public Health. 2021;45(2):129–32. 10.1111/1753-6405.13081 PMC8013313 33617133

[208] Pantoja-Melendez C, Garcia-De la Torre G, Duran-Robertson M, Peterson-Marquard K, Nunez-Amador S, Gomez-Bocanegra V, et al. COVID-19 Outbreak during summer courses at an elementary school. Children (Basel). 2023;10(3):418. 10.3390/children10030418 PMC10047848 36979976

[209] Chen D, Lau YC, Xu XK, Wang L, Du Z, Tsang TK, et al. Inferring time-varying generation time, serial interval, and incubation period distributions for COVID-19. Nat Commun. 2022;13(1):7727. 10.1038/s41467-022-35496-8 PMC9747081 36513688

[210] Rajendrakumar AL, Nair ATN, Nangia C, Chourasia PK, Chourasia MK, Syed MG, et al. Epidemic landscape and forecasting of SARS-CoV-2 in India. J Epidemiol Glob Health. 2021;11(1):55–9. 10.2991/jegh.k.200823.001 PMC7958270 32959618

[211] Ma S, Zhang J, Zeng M, Yun Q, Guo W, Zheng Y, et al. Epidemiological parameters of COVID-19: case series study. J Med Internet Res. 2020;22(10):e19994. 10.2196/19994 PMC7553786 33001833

[212] Huang S, Li J, Dai C, Tie Z, Xu J, Xiong X, et al. Incubation period of coronavirus disease 2019: new implications for intervention and control. Int J Environ Health Res. 2022;32(8):1707–15. 10.1080/09603123.2021.1905781 33818217

[213] Lee H, Kim K, Choi K, Hong S, Son H, Ryu S. Incubation period of the Coronavirus Disease 2019 (COVID-19) in Busan, South Korea. J Infect Chemother. 2020;26(9):1011–13. 10.1016/j.jiac.2020.06.018 PMC7311919 32631735

[214] Tomie T. Incubation period of COVID-19 in the “live-house” cluster of accurately known infection events and delay time from symptom onset of public reporting observed in cases in Osaka, Japan. medRxiv. 2020.

[215] Singh G, Patrikar S, Sankara Sarma P, Soman B. Time-dependent dynamic transmission potential and instantaneous reproduction number of COVID-19 pandemic in India. medRxiv. 2020.

[216] Nishiura H, Linton NM, Akhmetzhanov AR (2020). Serial interval of Novel Coronavirus (COVID-19) infections. Int J Infect Dis.

[217] Knight J, Mishra S (2020). Estimating effective reproduction number using generation time versus serial interval, with application to COVID-19 in the Greater Toronto Area, Canada. Infect Dis Model.

[218] Cai W, Yang Z, Liang J, Lin Z, Ma Y, Chen C, et al. How fast and how well the Omicron epidemic was curtailed. A Guangzhou experience to share. Front Public Health. 2022;10:979063. 10.3389/fpubh.2022.979063 PMC9812567 36620243

[219] Wang Y, Chen R, Hu F, Lan Y, Yang Z, Zhan C, et al. Transmission, viral kinetics and clinical characteristics of the emergent SARS-CoV-2 Delta VOC in Guangzhou, China. EClinicalMedicine. 2021;40:101129. 10.1016/j.eclinm.2021.101129 PMC8435265 34541481

[220] Abbott S, Sherratt K, Gerstung M, Funk S. Estimation of the test to test distribution as a proxy for generation interval distribution for the Omicron variant in England. medRxiv. 2022.

[221] Hao J, Hu XC, Fan MX, Chen J, Cheng QR, Li Z, et al. Analysis of clinical characteristics of 66 pediatric patients with B.1.617.2 (Delta) variant of COVID-19. World J Pediatr. 2022;18(5):343–49. 10.1007/s12519-022-00529-1 PMC8918891 35287229

[222] Grant R, Charmet T, Schaeffer L, Galmiche S, Madec Y, Von Platen C, et al. Impact of SARS-CoV-2 Delta variant on incubation, transmission settings and vaccine effectiveness: results from a nationwide case-control study in France. Lancet Reg Health Eur. 2022;13:100278. 10.1016/j.lanepe.2021.100278 PMC8616730 34849500

[223] Weil AA, Luiten KG, Casto AM, Bennett JC, O’Hanlon J, Han PD, et al. Mapping the emergence of SARS-CoV-2 Omicron variants on a university campus. medRxiv. 2022. 10.1038/s41467-022-32786-z PMC9446629 36068236

[224] Cortés Martínez J, Pak D, Abelenda-Alonso G, Langohr K, Ning J, Rombauts A, et al. SARS-Cov-2 incubation period according to vaccination status during the fifth COVID-19 wave in a tertiary-care center in Spain: a cohort study. BMC Infect Dis. 2022;22(1):1–7. 10.1186/s12879-022-07822-4 PMC9645305 36352359

[225] Han YN, Feng ZW, Sun LN, Ren XX, Wang H, Xue YM, et al. A comparative-descriptive analysis of clinical characteristics in 2019-coronavirus-infected children and adults. J Med Virol. 2020;92(9):1596–602. 10.1002/jmv.25835 32249943

[226] Tian S, Hu N, Lou J, Chen K, Kang X, Xiang Z, et al. Characteristics of COVID-19 infection in Beijing. J Infect. 2020;80(4):401–6. 10.1016/j.jinf.2020.02.018 PMC7102527 32112886

[227] Shiel E, Miyakis S, Tennant E, Fernando S, Kizny-Gordon A, Koh B, et al. Clinical characteristics and outcomes of COVID-19 in a low-prevalence, well resourced setting, Sydney, Australia. Intern Med J. 2021;51(10):1605–13. 10.1111/imj.15445 PMC8447053 34228387

[228] Guan W, Ni Z, Hu Y, Liang W, Ou C, He J, et al. Clinical characteristics of coronavirus disease 2019 in China. N Engl J Med. 2020;382(18):1708–20. 10.1056/NEJMoa2002032 PMC7092819 32109013

[229] Alsofayan YM, Althunayyan SM, Khan AA, Hakawi AM, Assiri AM. Clinical characteristics of COVID-19 in Saudi Arabia: A national retrospective study. J Infect Public Health. 2020;13(7):920–25. 10.1016/j.jiph.2020.05.026 PMC7833063 32534945

[230] Wang Y, Liao B, Guo Y, Li F, Lei C, Zhang F, et al. Clinical characteristics of patients infected with the Novel 2019 Coronavirus (Sars-Cov-2) in Guangzhou, China. Open Forum Infect Dis. 2020;7(6):ofaa187. 10.1093/ofid/ofaa187 PMC7299522 32577426

[231] Yang J, Wu K, Ding A, Li L, Lu H, Zhu W, et al. Clinical characteristics, treatment, and prognosis of 74 2019 Novel Coronavirus Disease patients in Hefei: a single-center retrospective study. Medicine (Baltimore). 2021;100(21):e25645. 10.1097/MD.0000000000025645 PMC8154399 34032692

[232] Pongpirul WA, Wiboonchutikul S, Charoenpong L, Panitantum N, Vachiraphan A, Uttayamakul S, et al. Clinical course and potential predictive factors for pneumonia of adult patients with Coronavirus Disease 2019 (COVID-19): a retrospective observational analysis of 193 confirmed cases in Thailand. PLoS Negl Trop Dis. 2020;14(10):1–17. 10.1371/journal.pntd.0008806 PMC7592908 33064734

[233] Song YS, Hao YB, Liu WW, Zhang SS, Wang P, Fan TL. Clinical features of 17 patients with 2019-nCoV. Eur Rev Med Pharmacol Sci. 2020;24(20):10896–901. 10.26355/eurrev_202010_23454 33155253

[234] Sun L, Shen L, Fan J, Gu F, Hu M, An Y, et al. Clinical features of patients with Coronavirus Disease 2019 from a designated hospital in Beijing, China. J Med Virol. 2020;92(10):2055–66. 10.1002/jmv.25966 PMC7267635 32369208

[235] Tan WYT, Wong LY, Leo YS, Toh MPHS. Does incubation period of COVID-19 vary with age? A study of epidemiologically linked cases in Singapore. Epidemiol Infect. 2020;148:e197. 10.1017/S0950268820001995 PMC7484300 32873357

[236] Qian GQ, Yang NB, Ding F, Ma AHY, Wang ZY, Shen YF, et al. Epidemiologic and clinical characteristics of 91 hospitalized patients with COVID-19 in Zhejiang, China: a retrospective, multicentre case series. QJM. 2020;113(7):474–81. 10.1093/qjmed/hcaa089 PMC7184349 32181807

[237] Chen G, Wu MZ, Qin CJ, Wu BB, Luo WR, Liu L, et al. Epidemiological analysis of 18 patients with COVID-19. Eur Rev Med Pharmacol Sci. 2020;24(23):12522–6. 10.26355/eurrev_202012_24049 33336772

[238] Yi B, Fen G, Cao D, Cai Y, Qian L, Li W, et al. Epidemiological and clinical characteristics of 214 families with COVID-19 in Wuhan, China. Int J Infect Dis. 2021;105:113–9. 10.1016/j.ijid.2021.02.021 PMC7872852 33578019

[239] Liu H, Gao J, Wang Y, Jie J, Luo J, Xu Y, et al. Epidemiological and clinical characteristics of 2019 Novel Coronavirus Disease (COVID-19) in Jilin, China: a descriptive study. Medicine (Baltimore). 2020;99(47):e23407. 10.1097/MD.0000000000023407 PMC7676609 33217886

[240] Liao J, Fan S, Chen J, Wu J, Xu S, Guo Y, et al. Epidemiological and clinical characteristics of COVID-19 in adolescents and young adults. Innovation (Camb). 2020;1(1):100001. 10.1016/j.xinn.2020.04.001 PMC7237935 33554183

[241] Chen W, Hu C, Huang L, Cai M, Zhang Y, Wei H, et al. Epidemiological and clinical characteristics of COVID-19 patients in Nanjing. Research Square. 2020.

[242] Gao Y, Ma X, Bi J, Chu J, Liu B, Chi C, et al. Epidemiological and clinical differences of Coronavirus Disease 2019 patients with distinct viral exposure history. Virulence. 2020;11(1):1015–23. 10.1080/21505594.2020.1802870 PMC7549930 32787496

[243] Liu P, Niu R, Chen J, Tang Y, Tang W, Xu L, et al. Epidemiological and clinical features in patients with Coronavirus Disease 2019 outside of Wuhan, China: special focus in asymptomatic patients. PLoS Negl Trop Dis. 2021;15(3):e0009248. 10.1371/journal.pntd.0009248 PMC7984620 33690662

[244] Deng L, Li Z, Liu Y, Li X, Jia N, Cui W, et al. Epidemiological and clinical findings of discharge patients infected with the 2019 novel coronavirus (SARS-COV-2) in Changchun, Northeast China: a retrospective cohort study. Acta Medica Mediterr. 2021;37(2):1147–53.

[245] Alhamlan FS, Almaghrabi RS, Devol EB, Alotaibi AB, Alageel SM, Obeid DA (2022). Epidemiology and clinical characteristics of people with confirmed SARS-CoV-2 infection during the early COVID-19 pandemic in Saudi Arabia. Medicines (Basel).

[246] Pung R, Chiew CJ, Young BE, Chin S, Chen MIC, Clapham HE, et al. Investigation of three clusters of COVID-19 in Singapore: implications for surveillance and response measures. Lancet. 2020;395(10229):1039–46. 10.1016/S0140-6736(20)30528-6 PMC7269710 32192580

[247] Ejima K, Kim KS, Ludema C, Bento AI, Iwanami S, Fujita Y, et al. Estimation of the incubation period of COVID-19 using viral load data. Epidemics. 2021;35:100454. 10.1016/j.epidem.2021.100454 PMC7959696 33773195

[248] Shen Q, Guo W, Guo T, Li J, He W, Ni S, et al. Novel coronavirus infection in children outside of Wuhan, China. Pediatr Pulmonol. 2020;55(6):1424–9. 10.1002/ppul.24762 PMC7262205 32259403

[249] Yang N, Shen Y, Shi C, Ma AHY, Zhang X, Jian X, et al. In-flight transmission cluster of COVID-19: a retrospective case series. Infect Dis (Lond). 2020;52(12):891–901. 10.1080/23744235.2020.1800814 32735163

[250] Ai J-W, Chen J-W, Wang Y, Liu X-Y, Fan W-F, Qu G-J, et al. The cross-sectional study of hospitalized Coronavirus Disease 2019 patients in Xiangyang, Hubei province. medRxiv. 2020.

[251] Qiu C, Deng Z, Xiao Q, Shu Y, Deng Y, Wang H, et al. Transmission and clinical characteristics of Coronavirus Disease 2019 in 104 outside-Wuhan patients, China. J Med Virol. 2020;92(10):2027–35. 10.1002/jmv.25975 PMC7267432 32369217

[252] Varghese B, Shajahan S, Anilkumar H, Haridasan RK, Rahul A, Thazhathedath H, et al. Symptomatology and epidemiologic characteristics of COVID 19 patients in Kerala, India. J Evol Med Dent Sci. 2020;9(46):3411–7.

[253] Xie S, Zhang G, Yu H, Wang J, Wang S, Tang G, et al. The epidemiologic and clinical features of suspected and confirmed cases of imported 2019 Novel Coronavirus pneumonia in North Shanghai, China. Ann Transl Med. 2020;8(10):637–7. 10.21037/atm-20-2119 PMC7290637 32566574

[254] Xu ZQ, Wu ZJ, Xu QB, Fang L, Cao XY, Chen WH, et al. The epidemiological characteristics of clusters of novel coronavirus pneumonia in Chenzhou, China. Acta Medica Mediterr. 2021;37(4):2187–9.

[255] Chen J, Zhang ZZ, Chen YK, Long QX, Tian WG, Deng HJ, et al. The clinical and immunological features of pediatric COVID-19 patients in China. Genes Dis. 2020;7(4):535–41. 10.1016/j.gendis.2020.03.008 PMC7194810 32363222

[256] Breton V, Guiguet-Auclair C, Odoul J, Peterschmitt J, Ouchchane L, Gerbaud L. Population based survey of the COVID-19 outbreak in the Haut-Rhin department from January to April 2020. SSRN. 2020.

[257] Galmiche S, Cortier T, Charmet T, Schaeffer L, Chény O, von Platen C, et al. SARS-CoV-2 incubation period across variants of concern, individual factors, and circumstances of infection in France: a case series analysis from the ComCor study. Lancet Microbe. 2023;4(6):e409–e17. 10.1016/S2666-5247(23)00005-8 PMC10112864 37084751

[258] Lessler J, Reich NG, Brookmeyer R, Perl TM, Nelson KE, Cummings DA (2009). Incubation periods of acute respiratory viral infections: a systematic review. Lancet Infect Dis.

[259] Vink MA, Bootsma MC, Wallinga J (2014). Serial intervals of respiratory infectious diseases: a systematic review and analysis. Am J Epidemiol.

[260] Assiri A, McGeer A, Perl TM, Price CS, Al Rabeeah AA, Cummings DA (2013). Hospital outbreak of Middle East respiratory syndrome coronavirus. N Engl J Med.

[261] Roll U, Yaari R, Katriel G, Barnea O, Stone L, Mendelson E (2011). Onset of a pandemic: characterizing the initial phase of the swine flu (H1N1) epidemic in Israel. BMC Infect Dis.

[262] Cauchemez S, Fraser C, Van Kerkhove MD, Donnelly CA, Riley S, Rambaut A (2014). Middle East respiratory syndrome coronavirus: quantification of the extent of the epidemic, surveillance biases, and transmissibility. Lancet Infect Dis.

[263] Asiedu-Bekoe F, Adu DA, Offei A (2012). Mass oseltamivir prophylaxis halts pandemic influenza A H1N1 2009 outbreak in a secondary school in Ashanti Region, Ghana. Ghana Med J.

[264] Sun K, Wang W, Gao L, Wang Y, Luo K, Ren L, et al. Transmission heterogeneities, kinetics, and controllability of SARS-CoV-2. Science. 2021;371(6526):eabe2424. 10.1126/science.abe2424 PMC7857413 33234698

[265] Puhach O, Meyer B, Eckerle I (2023). SARS-CoV-2 viral load and shedding kinetics. Nat Rev Microbiol.

[266] Shuai H, Chan JF, Hu B, Chai Y, Yuen TT, Yin F, et al. Attenuated replication and pathogenicity of SARS-CoV-2 B.1.1.529 Omicron. Nature. 2022;603(7902):693–9. 10.1038/s41586-022-04442-5 35062016

[267] Costa Clemens SA, Weckx L, Clemens R, Almeida Mendes AV, Ramos Souza A, Silveira MBV (2022). Heterologous versus homologous COVID-19 booster vaccination in previous recipients of two doses of CoronaVac COVID-19 vaccine in Brazil (RHH-001): a phase 4, non-inferiority, single blind, randomised study. Lancet.

[268] Buckner CM, Kardava L, El Merhebi O, Narpala SR, Serebryannyy L, Lin BC, et al. Interval between prior SARS-CoV-2 infection and booster vaccination impacts magnitude and quality of antibody and B cell responses. Cell. 2022;185(23):4333–46.e14. 10.1016/j.cell.2022.09.032 PMC9513331 36257313

[269] WHO Coronavirus (COVID-19) Dashboard for Vaccination. https://covid19.who.int/?mapFilter=vaccinations. Accessed 20 Apr 2023.

[270] Jiehao C, Jin X, Daojiong L, Zhi Y, Lei X, Zhenghai Q (2020). A case series of children with 2019 Novel Coronavirus infection: clinical and epidemiological features. Clin Infect Dis.

[271] Deeks JJ HJ, Altman DG. Chapter 10: Analysing data and undertaking meta-analyses. In: Higgins JPT, Thomas J, Chandler J, Cumpston M, Li T, Page MJ, Welch VA, editors. Cochrane Handbook for Systematic Reviews of Interventions version 6.3 (updated February 2022). Cochrane; 2022.

