# Supplementary material for: Assessing changes in incubation period, serial interval, and generation time of SARS-CoV-2 variants of concern: a systematic review and meta-analysis
Source: BMC Med. 2023 Sep 29;21:374. doi: 10.1186/s12916-023-03070-8 (PMC10541713; doi:10.1186/s12916-023-03070-8)
Supplement: Supplementary file 1 — Additional file 1: Table S1. Search strategy and search results. Table S2. Quality assessment scale. Table S3. Excluded studies and reason for exclusion. Table S4. Quality assessment of the studies used in the meta-analysis. Figure S1. Forest plot for studies of reporting estimates of the incubation period for different SARS-CoV-2 lineages. Figure S2. Funnel plot for the incubation period with a 95% CI for studies included in the meta-analysis. Figure S3. Forest plot for studies of reporting estimates of the serial interval for different SARS-CoV-2 lineages. Figure S4. Funnel plot for the serial interval with a 95% CI for studies included in the meta-analysis. Figure S5. Forest plot for studies of reporting estimates of the realized generation time for different SARS-CoV-2 lineages. Figure S6. Funnel plot for the realized generation time with a 95% CI for studies included in the meta-analysis. Figure S7. Forest plot for studies of reporting estimates of the intrinsic generation time for different SARS-CoV-2 lineages. Table S5. Characteristics of previous meta-analyses of the incubation period and/or serial interval of SARS-CoV-2 VOCs. [file 12916_2023_3070_MOESM1_ESM.pdf]

Supplementary Information for:

Assessing changes in incubation period, serial interval, and generation time of SARS-CoV-2 variants of concern: a systematic review and meta-analysis

Xiangyanyu Xu[1,&], Yanpeng Wu[2,&], Allisandra G. Kummer[3,&], Yuchen Zhao[2], Zexin Hu[2], Yan Wang[1], Hengcong Liu[1], Marco Ajelli[3,*,#], Hongjie Yu[1,2,*,#]

[1] School of Public Health, Fudan University, Key Laboratory of Public Health Safety, Ministry of Education, Shanghai, China

[2] Shanghai Institute of Infectious Disease and Biosecurity, Fudan University, Shanghai, China

[3] Laboratory of Computational Epidemiology and Public Health, Department of Epidemiology and Biostatistics, Indiana University School of Public Health, Bloomington, IN, USA

[&]Joint first authors

*Joint senior authors

[#]Corresponding authors: Marco Ajelli, marco.ajelli@gmail.com, Hongjie Yu, yhj@fudan.edu.cn

Table S1. Search strategy and search results.

Database	Step	Search strategy	Number of articles*
PubMed	#1	COVID-19 OR SARS-CoV-2 OR 2019-nCoV OR "coronavirus disease 2019" OR "severe acute respiratory syndrome coronavirus 2"	348,819
	#2	("serial interval*" OR "generation time*" OR "generation interval*") AND (contact tracing OR household)	196
	#3	"incubation period*"	15,603
	#4	English [Language]	30,661,800
	#5	#1 AND #2 AND #4	108
	#6	#1 AND #3 AND #4	725
	#7	#5 OR #6	790
Embase	#1	(COVID-19 OR SARS-CoV-2 OR 2019-nCoV OR "coronavirus disease 2019" OR "severe acute respiratory syndrome coronavirus 2") AND ((("serial interval*" OR "generation time*" OR "generation interval*") AND (contact tracing OR household)) OR "incubation period*")	864
Web of Science	#1	TS = (COVID-19 OR SARS-CoV-2 OR 2019-nCoV OR "coronavirus disease 2019" OR "severe acute respiratory syndrome coronavirus 2")	520,222
	#2	TS = (("serial interval*" OR "generation time*" OR "generation interval*") AND (contact tracing OR household))	263
	#3	TS = ("incubation period*")	40,802
	#4	#1 AND #2	137
	#5	#1 AND #3	1,167
	#6	#4 OR #5	1,244
	#7	#6 AND Language:English	1,058

Database	Step	Search strategy	Number of articles*
Europe PMC	#1	(COVID-19 OR SARS-CoV-2 OR 2019-nCoV OR "coronavirus disease 2019" OR "severe acute respiratory syndrome coronavirus 2") AND ((("serial interval" OR "generation time" OR "generation interval" OR "serial intervals" OR "generation times" OR "generation intervals") AND (contact tracing OR household)) OR "incubation periods") AND (LANG:"eng" OR LANG:"en" OR LANG:"us")	4,273
medRxiv	#1	SARS-CoV-2 AND "serial interval*" AND contact tracing	250
	#2	SARS-CoV-2 AND "generation time*" AND contact tracing	8,502
	#3	SARS-CoV-2 AND "generation interval*" AND contact tracing	862
	#4	SARS-CoV-2 AND "incubation period*"	4,871
	#5	SARS-CoV-2 AND "serial interval*" AND household	150
	#6	SARS-CoV-2 AND "generation time*" AND household	3,691
	#7	SARS-CoV-2 AND "generation interval*" AND household	452
	#8	#1 OR #2 OR #3 OR #4 OR #5 OR #6 OR #7	11,403
bioRxiv	#1	SARS-CoV-2 AND "serial interval*" AND contact tracing	31
	#2	SARS-CoV-2 AND "generation time*" AND contact tracing	3,515
	#3	SARS-CoV-2 AND "generation interval*" AND contact tracing	77
	#4	SARS-CoV-2 AND "incubation period*"	5,940
	#5	SARS-CoV-2 AND "serial interval*"AND household	6
	#6	SARS-CoV-2 AND "generation time*" AND household	298
	#7	SARS-CoV-2 AND "generation interval*" AND household	17

Database	Step	Search strategy	Number of articles*
	#8	#1 OR #2 OR #3 OR #4 OR #5 OR #6 OR #7	7,398
arXiv	#1	COVID-19 OR SARS-CoV-2 OR 2019-nCoV OR "coronavirus disease 2019" OR "severe acute respiratory syndrome coronavirus 2"	7,123
	#2	"serial interval" OR "generation time" OR "generation interval" OR "incubation period" OR "serial intervals" OR "generation times" OR "generation intervals" OR "incubation periods"	1,169
	#3	#1 AND #2	56
SSRN	#1	COVID-19 AND "serial interval*"	14
	#2	COVID-19 AND "generation time*"	6
	#3	COVID-19 AND "generation interval*"	1
	#4	COVID-19 AND "incubation period*"	39
	#5	SARS-CoV-2 AND "serial interval*"	8
	#6	SARS-CoV-2 AND "generation time*"	5
	#7	SARS-CoV-2 AND "generation interval*"	1
	#8	SARS-CoV-2 AND "incubation period*"	13

*Searches were conducted on 28 March 2023.

Table S2. Quality assessment scale

A	**Robustness of data collection for contacts**<br>1. Includes contacts regardless of the clinical outcome, including individuals that test negative<br>2. Includes contacts from a specific setting (e.g., household) regardless of the clinical outcome, including individuals that test negative<br>3. Includes a subset of contacts that tested positive<br>4. No description/not clear
B	**Representativeness of the study cohort – Applies to all estimates**<br>1. No selection of cases based on age, sex, or general health status, supported by descriptive statistics demonstrating comparability with overall population<br>2. No selection of cases based on age, sex, or general health status, not supported by descriptive statistics<br>3. Cases are likely to be biased towards those with more severe COVID-19 symptoms due to selection process – e.g. records from hospitalized patients<br>4. Cases are selected (e.g. based on age or sex) to represent a particular cohort of individuals<br>5. No description of the derivation of the cohort
C	**Precision of the exposure window for cases used in final analysis – Applies to incubation period and generation time estimates**<br>1. Only includes cases with a 1-day exposure window<br>2. Only includes cases with less than or equal to 3-day exposure window<br>3. Includes cases with more than 3-day exposure window/unknown length of window<br>4. Includes cases with first day, last day or intermediate day of exposure window<br>5. No description/not clear
D	**Identification of potential infector(s) – Applies to all estimates**<br>1. Multiple exposures but statistical methods are applied to account for this<br>2. Multiple exposures but no statistical methods are applied to account for this<br>3. Single exposure<br>4. No description/not clear

E	**Precision of estimate of the symptom onset – Applies to incubation period and serial interval estimates**<br>1. Precise date<br>2. Window<br>3. No description/not clear
F	**Precision of estimate for symptom onset of identified potential infector(s) – Applies to serial interval estimate**<br>1. Precise date<br>2. Window<br>3. No description/not clear
G	**Distribution of the incubation period used in the analysis – Applies to generation time estimate**<br>1. Both the variant and location match those used in the estimation.<br>2. Either the variant or the location match those used in the estimation.<br>3. Neither the variant nor the location match those used in the estimation.<br>4. No description/not used

Table S3. Excluded studies and reason for exclusion.

Studies	Reason for exclusion
[167-179]	Reviews
[11, 16, 17, 23, 180-194]	Meta-analyses
[195]	Comment
[196-199]	Full text unavailable
[200]	Study period unavailable
[201-205]	Sample size less than 5
[206-209]	Estimate unavailable
[210-217]	Data source unknown
[87, 218]	Methods not described
[219-256]	Unknown epidemiological link

Table S4. Quality assessment of the studies used in the meta-analysis

	Study	Quality Assessment						
		A	B	C	D	E	F	G
Ancestral Lineage	Shen et al., 2020[26]	1	1	4	4	1	NA	NA
	Vazirinejad et al., 2020[29]	1	1	NA	4	1	1	NA
	Bender et al., 2021[32]	1	1	3	4	1	1	4
	Liu et al., 2020[34]	3	1	5	4	1	1	NA
	Haddad et al., 2021[37]	1	1	NA	4	1	1	NA
	Shi et al., 2020[38]	1	1	5	4	1	1	NA
	Song et al., 2020[42]	2	1	5	4	3	NA	NA
	Gupta et al., 2020[49]	1	1	NA	4	1	1	NA
	Bao et al., 2021[53]	1	1	5	4	1	1	NA
	Du et al., 2020[56]	3	1	NA	4	1	1	NA
	Li et al., 2020[166]	3	1	5	4	1	1	NA
	Zhu et al., 2021[60]	3	1	3	4	1	1	NA
	Ping et al., 2021[64]	1	1	3	4	1	1	NA
	Ki, 2020[67]	1	1	5	4	1	1	NA
	Mao et al., 2020[70]	1	1	1	3	1	NA	NA
	Zhang et al., 2020[73]	1	1	5	4	1	NA	NA
	Guo et al., 2020[85]	3	4	5	4	1	NA	NA
	Nie et al., 2020[86]	3	1	4	4	1	NA	NA
	Son et al., 2020[89]	1	1	NA	4	1	1	NA
	Baskaradoss et al., 2021[90]	1	1	NA	4	1	1	NA
	Du et al., 2021[92]	3	1	4	4	1	NA	NA
	Thway et al., 2020[93]	3	1	NA	4	1	1	NA
	Kwok et al., 2020[94]	1	1	NA	4	1	1	NA
	Hua et al., 2020[97]	2	4	4	4	1	NA	NA
	Wong et al., 2020[98]	1	1	1	4	1	1	NA
	Haw et al., 2020[99]	3	1	NA	4	1	1	NA
	Ganyani et al., 2020[101]	3	2	5	4	NA	NA	2
	Hart et al., 2022[102]	2	1	5	1	NA	NA	1
	Hu et al., 2021[9]	1	1	4	4	NA	NA	2
	Zhao et al., 2021[103]	3	2	5	4	1	NA	4
	Lau et al., 2021[104]	3	2	3	4	1	NA	4
	Deng et al., 2021[105]	3	2	3	4	1	NA	NA
	Böhm et al., 2021[106]	1	1	3	3	1	1	NA
	Böhmer et al., 2020[107]	1	1	4	4	1	1	NA
	Yang et al., 2020[109]	3	1	4	3	1	1	NA
	Zhang et al., 2020[110]	3	1	3	3	1	1	NA
	Backer et al., 2020[111]	4	1	3	4	1	NA	NA
	Bui et al., 2020[112]	3	1	3	4	1	NA	NA
	Cheng et al., 2021[113]	4	1	3	4	1	NA	NA
	Han, 2020[114]	1	1	5	4	1	NA	NA

	Kong, 2020[115]	4	2	2	4	1	NA	NA
	Xiao et al., 2021[117]	4	1	5	4	1	NA	NA
	Xiao et al., 2020[118]	4	1	5	4	1	NA	NA
	Ratovoson et al., 2022[119]	2	1	3	4	1	1	NA
	Aghaali et al., 2020[120]	1	2	NA	4	3	3	NA
	Bi et al., 2020[121]	1	1	3	4	1	1	NA
	Expert Taskforce,2020[123]	1	1	NA	4	1	1	NA
	McAloon et al., 2021[124]	1	2	NA	4	3	3	NA
	Talmoudi et al., 2020[126]	3	2	NA	4	1	1	NA
	Wang et al., 2020[127]	3	1	NA	4	1	1	NA
	Kwok et al., 2021[128]	3	1	NA	3	1	1	NA
	Dai et al., 2020[130]	3	1	3	4	1	NA	NA
	Huang et al., 2020[131]	1	4	5	4	1	1	NA
	Areekal et al., 2021[133]	1	1	4	4	1	1	NA
	Tindale et al., 2020[136]	3	2	3	4	1	1	NA
	Won et al., 2021[137]	3	2	5	4	1	1	NA
	Xia et al., 2020[138]	3	1	2	4	1	1	NA
	Zhao et al., 2021[139]	1	1	4	4	1	1	NA
	Emecen et al., 2021[141]	3	3	3	3	1	1	NA
	Li et al., 2020[142]	3	2	4	4	1	1	4
	Ali et al., 2020[10]	3	2	NA	4	1	1	NA
	Bernal Lopez et al., 2022[143]	2	1	5	4	1	1	NA
	Cereda et al., 2021[144]	1	1	NA	4	1	1	NA
	Hong et al., 2020[145]	3	1	NA	4	3	3	NA
	Lavezzo et al., 2020[146]	2	1	NA	4	3	3	NA
	Liu et al., 2020[147]	3	2	NA	4	1	1	NA
	Najafi et al., 2020[148]	3	2	NA	4	1	1	NA
	Prete et al., 2020[149]	3	2	NA	4	1	1	NA
	Reed et al., 2021[150]	3	2	NA	4	1	1	NA
	Ryu et al., 2021[151]	3	2	NA	4	1	1	NA
	Saurabh et al., 2020[152]	3	2	NA	4	1	1	NA
	Thai et al., 2021[153]	1	2	NA	4	1	1	NA
	Qin et al., 2020[155]	4	1	5	4	1	NA	NA
	Ren et al., 2021[156]	3	1	3	4	1	1	NA
	Zhao et al., 2020[157]	3	2	NA	4	1	1	NA
	Adam et al., 2020[158]	3	2	NA	3	1	1	NA
	Ferretti et al., 2020[159]	3	2	5	4	NA	NA	2
	Althobaity et al., 2022[160]	3	2	3	4	1	1	NA
	Wang et al., 2022[161]	1	2	NA	4	1	1	NA
	Geismar et al., 2021[44]	2	1	NA	3	1	1	NA
	Buchholz et al., 2022[68]	2	2	NA	3	1	1	NA
	Somda et al., 2022 [164]	3	1	NA	4	3	3	NA
	Hammond et al., 2022 [165]	1	1	3	3	1	1	NA

	Geismar et al., 2022 [84]	2	2	NA	4	3	3	NA
Alpha	Hart et al., 2022 [22]	2	2	5	1	NA	NA	3
	Manica et al., 2023[21]	2	1	3	1	1	1	1
	Geismar et al., 2021[44]	2	1	NA	3	1	1	NA
	Tanaka et al., 2022[52]	1	1	1	3	1	NA	NA
	Amato et al., 2022[55]	3	1	3	2	NA	NA	3
	Buchholz et al., 2022[68]	2	2	NA	3	1	1	NA
	Geismar et al., 2022 [84]	2	2	NA	4	3	3	NA
Beta	Investigation team, 2021[69]	1	1	5	4	1	NA	NA
Delta	Hart et al., 2022 [22]	2	2	5	1	NA	NA	3
	Manica et al., 2023[21]	2	1	3	1	1	1	1
	Li et al., 2022[31]	3	1	1	4	1	1	NA
	Kang et al., 2022[39]	1	1	3	4	1	1	NA
	Ogata et al., 2022[40]	1	1	1	3	1	1	NA
	Pung et al., 2021[13]	2	2	NA	4	3	3	NA
	Ryu et al., 2022[45]	3	2	NA	2	1	1	NA
	Kremer et al., 2022[46]	3	2	NA	4	1	1	NA
	Backer et al., 2022[47]	3	2	3	3	1	1	NA
	Hwang et al., 2022[50]	3	1	NA	4	1	1	NA
	Li et al., 2022[51]	1	1	5	4	1	1	NA
	Song et al., 2022[57]	3	2	NA	4	1	1	NA
	McAleavey et al., 2022[58]	3	1	5	4	1	NA	NA
	Park et al., 2022[59]	3	2	3	3	1	1	NA
	Zhang et al., 2021[63]	3	2	1	4	3	3	1
	Liu et al., 2022[65]	4	2	3	4	1	NA	NA
	Buchholz et al., 2022[68]	2	2	NA	3	1	1	NA
	Ali et al., 2022[71]	3	2	NA	3	1	1	NA
	Zeng et al., 2023 [12]	3	1	1	3	1	1	NA
	Wang et al., 2022 [79]	3	1	NA	3	1	1	NA
	Ogata et al., 2023 [80]	3	1	1	4	1	NA	NA
	Luo et al., 2023 [81]	1	1	3	4	1	1	4
	Guo et al., 2023 [83]	3	3	5	4	1	1	NA
	Geismar, et al., 2022 [84]	2	2	NA	4	3	3	NA
Omicron	Song et al., 2022[27]	2	1	3	4	1	1	NA
	Manica et al., 2022[28]	2	1	1	1	1	1	2
	Mefsin et al., 2022[30]	3	2	1	4	1	1	4
	Brandal et al., 2021[43]	1	2	1	4	1	NA	NA
	Kremer et al., 2022[46]	3	2	NA	4	1	1	NA
	Backer et al., 2022[47]	3	2	3	3	1	1	NA
	Kim et al., 2022[48]	1	1	NA	4	1	1	NA
	Tanaka et al., 2022[52]	1	1	1	3	1	NA	NA
	Shim et al., 2022[54]	3	1	NA	4	1	1	NA

	Park et al., 2022[59]	3	2	3	3	1	1	NA
	HelmSDal et al., 2022[62]	1	2	5	4	1	NA	NA
	Liu et al., 2022[65]	4	2	3	4	1	NA	NA
	Buchholz et al., 2022[68]	2	2	NA	3	1	1	NA
	Ali et al., 2022[71]	3	2	NA	3	1	1	NA
	Zeng et al., 2023 [12]	3	1	1	3	1	1	NA
	Xiong et al., 2023 [74]	3	1	3	4	1	NA	NA
	Wei et al., 2023 [76]	2	3	3	3	3	3	NA
	Wang et al., 2023 [78]	1	1	3	3	1	NA	4
	Ogata et al., 2023 [80]	3	1	1	4	1	NA	NA
	Guo et al., 2023 [82]	3	1	NA	3	1	1	NA
	Guo et al., 2023 [83]	3	3	5	4	1	1	NA
	Geismar et al., 2022 [84]	2	2	NA	4	3	3	NA

Note: If a question does not apply (e.g., the study does not include generation time estimates), we classified it as NA.

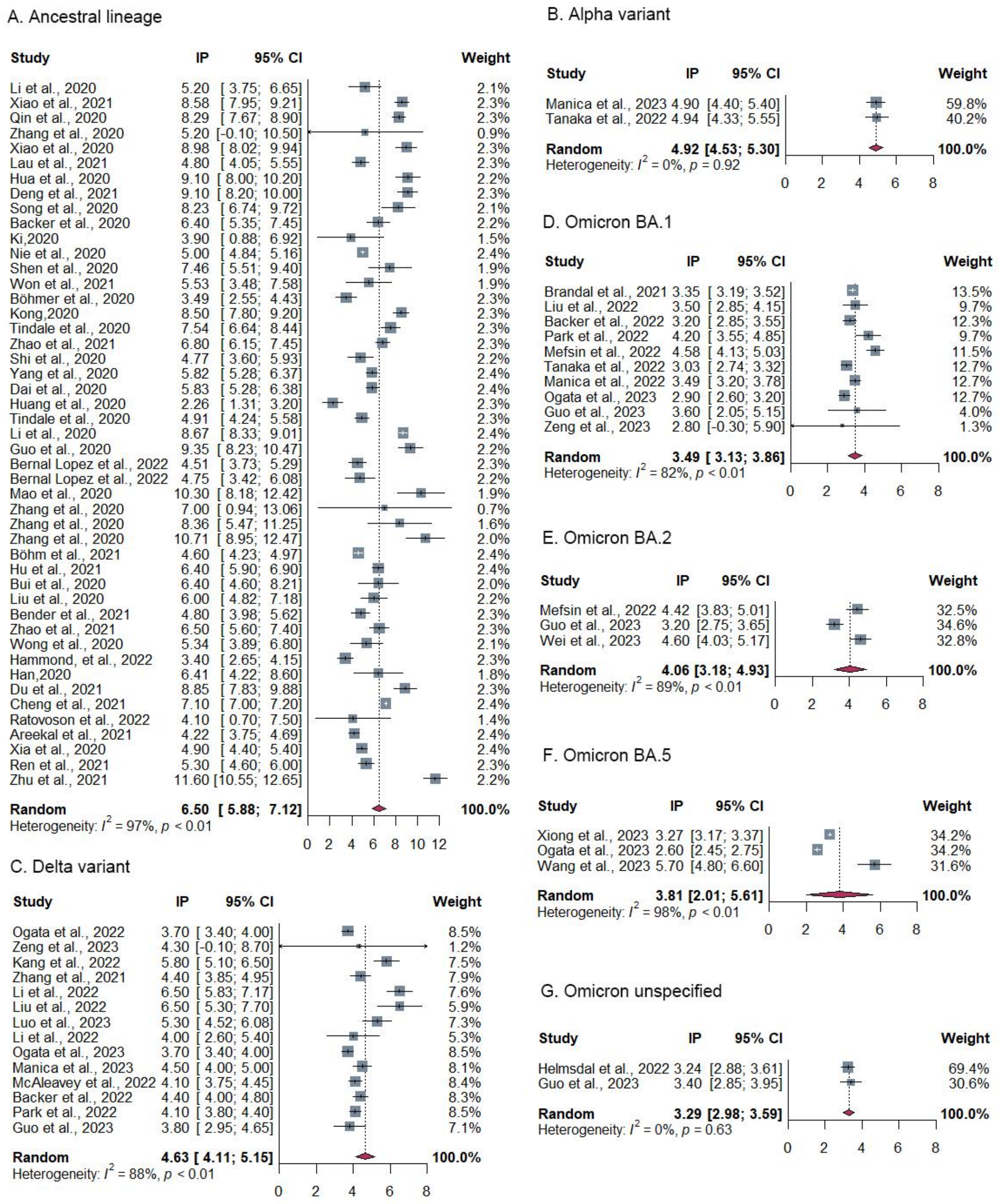

Figure S1. Forest plot for studies of reporting estimates of the incubation period for different SARS-CoV-2 lineages. Eighty-one records were selected and included into meta-analysis. Twenty-nine records were excluded since they did not provide a central or dispersion tendency estimate, nor could one be inferred from the available summary statistics. The Beta variant had only one record, thus it was unable to be included.

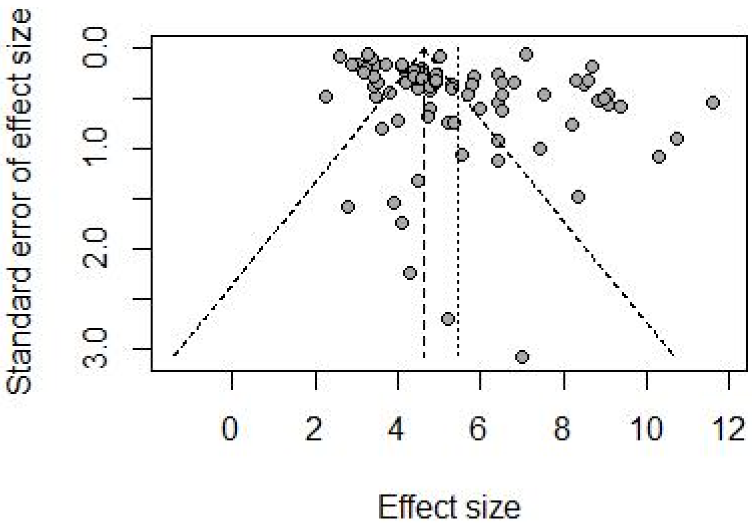

Figure S2. Funnel plot for the incubation period with a 95%CI for studies included in the meta-analysis.

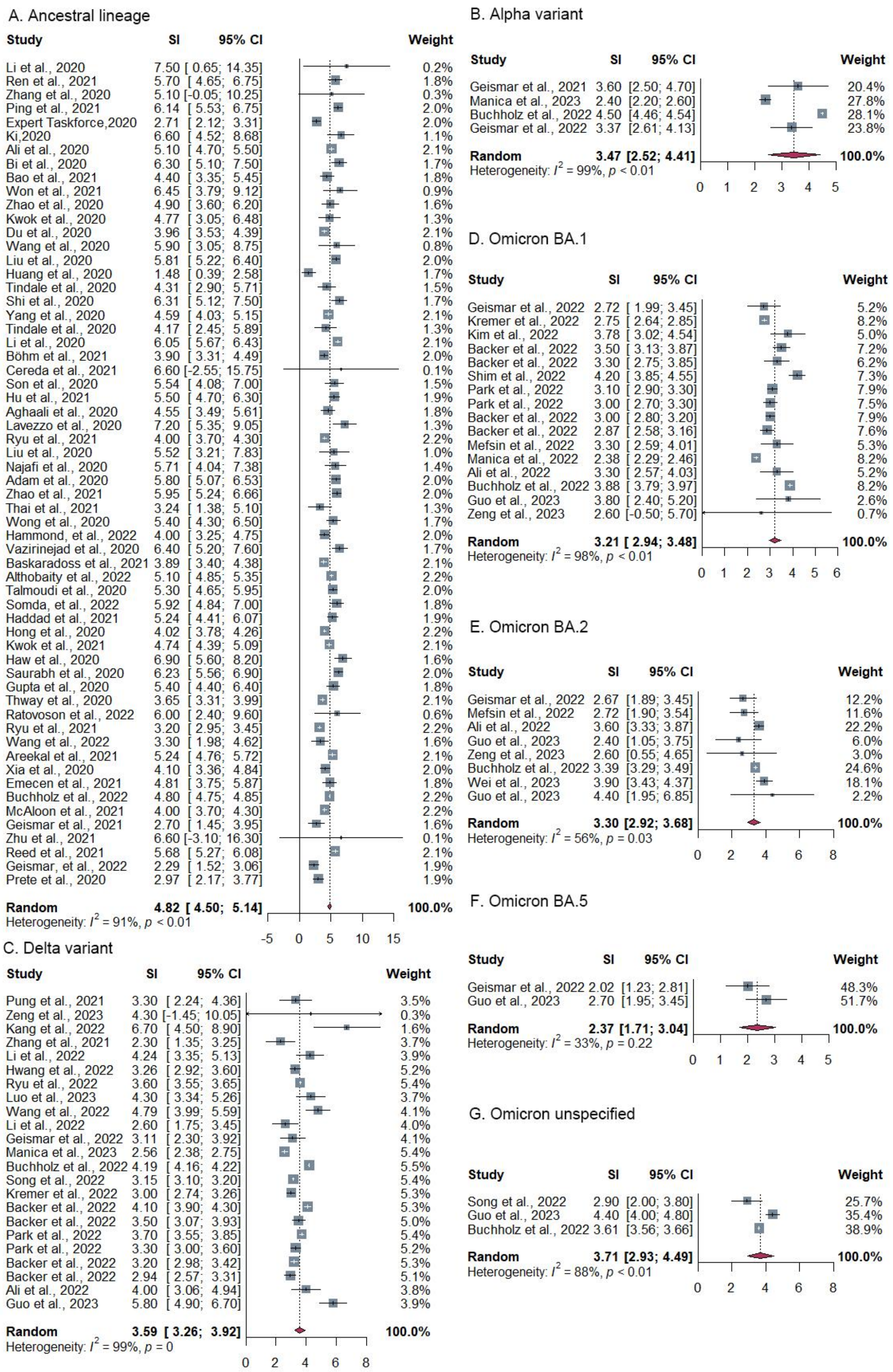

Figure S3. Forest plot for studies of reporting estimates of the serial interval for different SARS-CoV-2 lineages. One hundred and sixteen records were selected and included into

meta-analysis. Twenty records were excluded since they did not provide a central or dispersion tendency estimate, nor could one be inferred from the available summary statistics. Omicron BA.4 had only one record, thus it was unable to be included.

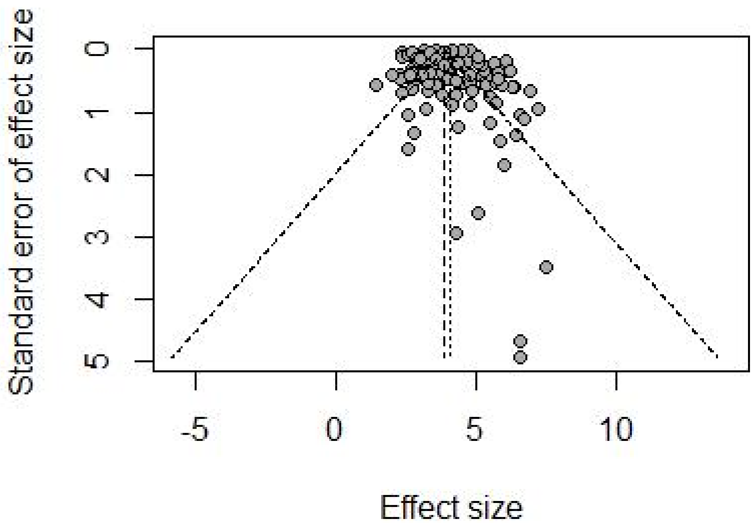

Figure S4. Funnel plot for the serial interval with a 95%CI for studies included in the meta-analysis.

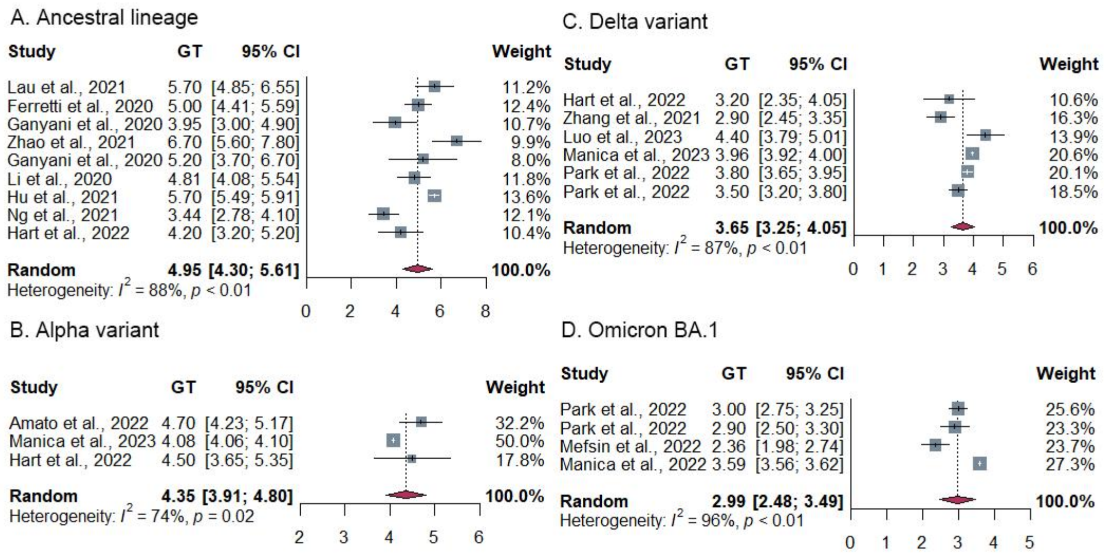

Figure S5. Forest plot for studies of reporting estimates of the realized generation time for different SARS-CoV-2 lineages. Twenty-two records were selected and included into meta-analysis. Four records were excluded since they did not provide a central or dispersion tendency estimate, nor could one be inferred from the available summary statistics. Omicron BA.5 had only one record, thus it was unable to be included.

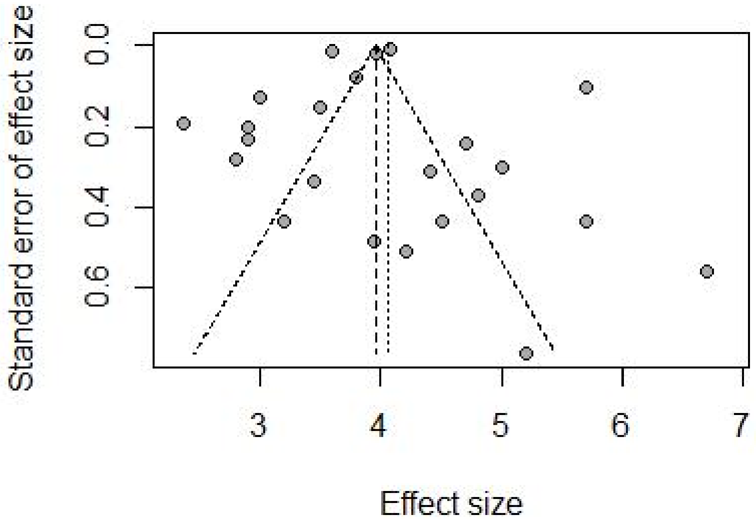

Figure S6. Funnel plot for the realized generation time with a 95%CI for studies included in

the meta-analysis.

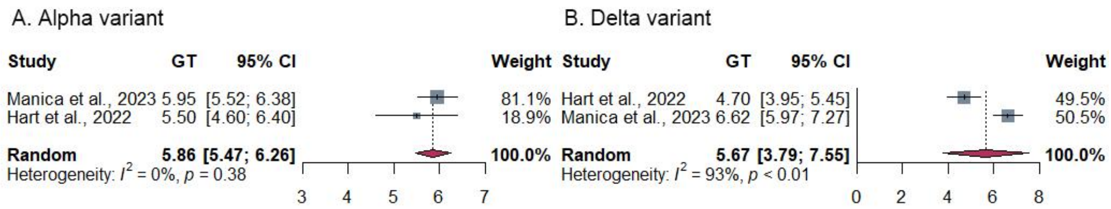

Figure S7. Forest plot for studies of reporting estimates of the intrinsic generation time for different SARS-CoV-2 lineages. Four records were selected and included into meta-analysis. Omicron BA.1 had only one record, thus it was unable to be included.

Table S5. Characteristics of previous meta-analyses of the incubation period and/or serial interval of SARS-CoV-2 VOCs.

Study	Parameter(s)	Lineages	Database(s)	Search time	Number of studies	Number of estimates
Du et al., 2022 [17]	Serial interval, incubation period	Alpha, Beta, Delta, Omicron	PubMed	Up to 10 March 2022	19	33
Wu et al., 2022 [16]	Incubation period	Ancestral lineage, Alpha, Beta, Delta, Omicron	PubMed, Embase and ScienceDirect	Up to 10 February 2022	142	142
Madewell et al., 2023 [18]	Serial interval	Delta, Omicron (disaggregated by BA1, BA2, BA5)	PubMed, Scopus, Cochrane Library, ScienceDirect, and medRxiv	Up to 23 May 2023	31	49
